# Comparison of three elements (In, Sn, and Sb) in the same period as catalysts in the ring-opening polymerization of l-lactide: from amorphous to semicrystalline polyesters†‡

**DOI:** 10.1039/d4ra06783e

**Published:** 2024-10-31

**Authors:** Oscar F. González-Belman, J. Oscar C. Jiménez-Halla, Gerardo González, José E. Báez

**Affiliations:** a Department of Chemistry, University of Guanajuato (UG) Noria Alta S/N 36050 Guanajuato Gto Mexico jebaez@ugto.mx

## Abstract

The ring-opening polymerization (ROP) of l-lactide (l-LA) is the main method for synthesizing poly(l-lactide) (PLLA), in which choosing the catalyst is one of the most important parameters. In this work, we focused on the systematic study of catalysts based on p-block elements from period 5, such as indium(iii), tin(ii), tin(iv) and antimony(iii) acetates, which displayed contrasting performances influenced by the oxidation state of the metal center. Analysis of the obtained oligomers by different techniques, including nuclear magnetic resonance (NMR), differential scanning calorimetry (DSC), polarized optical microscopy (POM) and matrix-assisted laser desorption ionization-time-of-flight (MALDI-TOF), revealed the selectivity of each catalyst toward the ROP of l-LA. Tin(ii) acetate showed the best performance, making it the best catalyst of this series for synthesizing PLLA. Indium(iii) and tin(ii) acetates induced an amorphous and semicrystalline polyester, respectively. The kinetic study evidenced the excellent performance of tin(ii) acetate in the ROP of l-LA. This catalyst reached high conversions in a quarter of the total reaction time, positioning it as the most catalytically active of the selected p-block acetate catalysts. Finally, the coordination-insertion mechanism by the catalyst in the initiation step was corroborated through the development of a mechanistic study applying the density functional theory (DFT).

## Abstract 2

This work explores the effect of three p-block elements (In, Sn, and Sb) in the same period of the periodic table as catalysts in the ring-opening polymerization of l-lactide.

## Graphical abstract

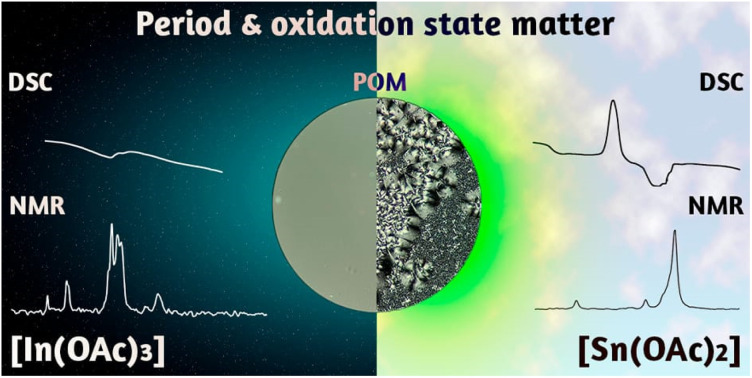

## Introduction

Nowadays, polymeric materials have become very important for our daily routine because several everyday products, such as clothes, packing materials, furniture, tools, coatings, and cosmetics, are made of polymers with optimal mechanical properties to satisfy the customer needs. Some of the main advantages of polymers (such as polyethylene) are their physical (thermoplastic) and chemical (stability to support acid and basic conditions) properties.^1^ However, this stability is also a significant problem in terms of biodegradation, contributing to the generation of residues and waste. In this sense, the accumulation of polymers in the environment promote (by radiation and oxidation) their fragmentation and generation of microplastics.^2,3^ In the last few years, biodegradable polyesters such as polylactic acid (PLA) have become one of the best candidates to substitute the petrol-based polymers due to their good processability, high mechanical strength, low thermal expansion, good adhesion, biodegradability, and biocompatibility.^4^ These interesting features of PLA are very attractive for industrial^5–10^ and medical applications.^11–15^ Conversely, polymers such as poly(l-lactide) (PLLA) are mostly obtained through the coordination-insertion process, where the design or choice of an active catalyst is the key to obtaining the desired polyester. Reports have described compounds of alkali metals,^16–19^ alkaline-earth metals,^20–27^ rare-earth elements,^28–34^ transition metals,^23,35–40^ and p-block elements^26,41–59^ as species with catalytic activity in the ring-opening opening polymerization (ROP) of l-lactide (l-LA). To find the catalyst with the best catalytic performance, previous works have involved the comparative study of ROP catalysts bearing different metal centers throughout a group,^22,37,40,60–62^ period^63^ or block^20,21,23–27,30–32,64,65^ of the periodic table (Table 1).

**Table 1:** Advantages and disadvantages of some metal-catalysts in the ROP of l-LA

Catalyst	Conv. (%)	Time	Polymerization control	Bulk or solvent	Side reactions
Na^16^^,^^a^	0–99	1–5^c^	✗	Solvent	✓
Mg^20^^,^^a^	97–100	5–10^c^	✓	Solvent	✗
Zn^25^^,^^a^	77–93	3–60^c^	✗	Bulk	✓
Gd^29^^,^^a^	89–90	8–24^d^	✓	Solvent	✗
Sm^30^^,^^b^	77	5^c^	✗	Solvent	✓
In^42^^,^^b^	95–97	2–4.5^d^	✗	Bulk	✗

a In the presence of an initiator.

b In the absence of an initiator.

c Minutes.

d Hours.

Of all the studied catalysts, the ones based on the tin element have shown the best performance in the ROP of l-LA since most of these compounds can catalyze the polymerization under bulk or solvent conditions with excellent conversion efficiency.^50,53,57,66–68^ Focusing on the placement of tin in the periodic table, there has been a significant number of published contributions on their derivatives such as tin(ii) octoate,^58^ tin(ii) guanidinate,^50^ and tin(ii) chloride.^57^ Although indium and antimony are also present in the same period, there are very few studies involving them in the ROP of l-LA. The introduction of indium and antimony to our study will provide a better understanding of the catalytic activity throughout the period.

**Scheme 1:**
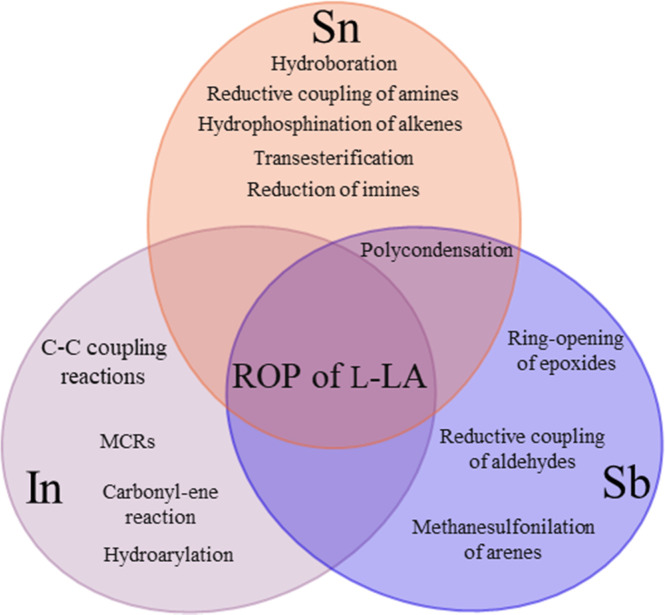
Different chemical reactions assisted by indium, tin, and antimony compounds.

In previous contributions, different authors have reported on the catalytic exploration of compounds bearing p-block elements belonging to period 5, such as indium, tin and antimony (Scheme 1), which can catalyze a wide range of transformations, such as multicomponent reactions,^69^ C–C bond formation,^70–72^ hydroarylation,^71^ carbonyl–ene,^71^ hydroboration,^73^ reductive coupling of amines,^74^ hydrophosphination of alkenes,^75^ transesterification,^76^ reduction of imines,^77^ hydrolysis of triacylglyceridecin,^78^ methanesulfonylation of arenes,^79^ reductive coupling of aldehydes,^80^ ring-opening of epoxides,^81,82^ polycondensation,^83,84^ among others. In this work, we focus on a systematic study of indium(iii), tin(ii), tin(iv) and antimony(iii) acetates as catalysts for the ROP of l-LA under bulk conditions,^59^ which have advantages, including solvent-free conditions, high temperatures, and no inert atmosphere.

## Experimental

### Materials

All reagents, including l-lactide (l-LA, purity 98%), indium(iii) acetate (purity 99.99%), tin(ii) acetate (purity ≤100%), tin(iv) acetate (purity ≤100%), antimony(iii) acetate (purity 99.99%), ethylene glycol (purity ≥99.5%), 1-octanol (purity ≥99%) and 1,8-octanediol (purity 98%), were purchased from Sigma-Aldrich and used as received.

### Ring-opening polymerization (ROP) of l-lactide (l-LA)

The bulk ROP reactions were carried out by adding the components in this order: monomer (20 mmol, l-LA), initiator (2 mmol, water, ethylene glycol, 1-octanol or 1,8-octanediol) and catalyst [0.03 mmol, indium(iii), tin(ii), tin(iv) and antimony(iii) acetates] in a dried vial. Then, the mixture was heated at 140 °C with constant stirring (at 220 rpm) for 80 min. Once the reaction time finished, the crude product of the reaction was analyzed by NMR to collect the evidence of the polymerization process. In some cases, the obtained PLLAs were precipitated from chloroform/methanol, recovered by filtration, and dried under vacuum. The number-average molecular weight (*M*_n_) and conversion were monitored by ^1^H NMR spectroscopy. In the case of the sample entry 10 (Table 4): ^1^H NMR (500 MHz, CDCl_3_, ppm, Fig. S1‡): *δ* 5.17 (d, multiplet, 1H, [CH(CH_3_)–O–], PLLA), 5.03 (quadruplet, 1H, [O=CH(CH_3_)–O], l-LA), 4.35 (e, quadruplet, 1H, [–CH(CH_3_)–OH], PLLA), 4.12 (c, multiplet, 2H, [–CH_2_–O–CO], PLLA), 1.67 (doublet, 3H, [O=CH(CH_3_)–O], l-LA), 1.58 (g, triplet, 3H, [–CH(CH_3_)–O–], PLLA), 1.50 (f, doublet, 3H, [O=CH(CH_3_)–O], PLLA), 1.49 (h, doublet, 3H, [–CH(CH_3_)–OH], PLLA), 1.28 (b, multiplet, 2H, [–CH_2_–], PLLA), 0.87 (a, triplet, 3H, [–CH_2_–CH_3_], PLLA). ^13^C NMR (500 MHz, CDCl_3_, ppm, Fig. S2‡): ^1^CH_3_–^2^CH_2_–^3^CH_2_–^4^CH_2_–^5^CH_2_–^6^CH_2_–^7^CH_2_–^8^CH_2_–O–^1^CO–^10^CH(^11^CH_3_)–O–[^12^CO–^13^CH(^14^CH_3_)–O]_*n*−1_–^15^CO–^16^CH(^17^CH_3_)–OH: *δ* 175.28 (1), 170.29 (15), 169.74 (12), 167.45 (l-LA), 77.61 (l-LA), 69.47 (10), 69.15 (13), 66.86 (16), 65.84 (8), 31.88 (3), 29.26 (5), 29.25 (4), 28.60 (7), 25.87 (6), 22.75 (2), 20.66 (17), 16.97 (11), 16.79 (14), 15.97 (l-LA), 14.20 (1). IR (cm^−1^) (Fig. 4): 3454 (O–H, *ν*), 2991 (CH_3_, *ν*_as_) 2940 (CH_2_ and CH *ν*_as_), 2859 (CH_3_, *ν*_s_), 1738 (C=O, *ν*), 1453 (CH_3_, *δ*_as_), 1187 (–C–(CO)–O–, *ν*_as_), 1090 (–C–O–C–, *ν*). DSC (Fig. S3A‡): *T*_g_ = 14 °C, *T*_c_ = 71 °C (Δ*H*_c_ = 24 J g^−1^), *T*_m_ = 114 °C (Δ*H*_m_ = 24 J g^−1^). *M*_n NMR_ = 1530, *M*_n MALDI_ = 1035, *Đ*_M_ = 1.03.

### Characterization methods

FT-IR analysis was carried out on a PerkinElmer Spectrum 100 FT-IR spectrometer with attenuated total reflectance spectroscopy (ATR) accessory. The ^1^H and ^13^C NMR spectra were obtained at room temperature on a 500 MHz Bruker Avance III HD instrument, using CDCl_3_ as solvent. The percent of conversion [conv. (%)_NMR_] obtained by ^1^H NMR was calculated according to the equation: conv. (%) = (*I*_pol_/#*H*_pol_)/[(*I*_pol_/#*H*_pol_) + (*I*_mon_/#*H*_mon_)] × 100. The degree of polymerization (DP_NMR_) was quantified by ^1^H NMR using the following equation: DP = (*I*_pol_/#*H*_pol_)/(*I*_gter_/#*H*_gter_) + 1; where *I*_pol_ is the integral value of the methine (–CH–) in the repetitive unit of the polymer at 5.16 ppm, *I*_mon_ is the integral value of the methine (–CH–) of the monomer at 5.03 ppm, *I*_gter_ is the integral value of the terminal group, corresponding to methine attached to hydroxyl (–CH–OH, *δ* 4.35) or methyl end-group (–CH_3_, *δ* 0.87), and the number 1 is the contribution of the terminal group. #*H*_pol_, #*H*_mon_, and #*H*_gter_ represent the number of protons associated with the polymer, monomer and terminal group, respectively.

The matrix assisted laser desorption ionization-time-of-flight (MALDI-TOF) spectra were obtained in a Bruker Microflex instrument that incorporates a nitrogen laser with a wavelength of 337 nm in positive polarity accelerated at 20 kV in reflector mode. The preparation of the samples and the matrix (dithranol) were carried out by their dissolution in THF and water/acetonitrile/trifluoroacetic acid solution (60/40/0.1), respectively. Then, 2 μL of the sample solution were mixed with 5 μL of the matrix solution. From the resulting mixture, 2 μL was taken and placed in a stainless-steel plate for 20 minutes to evaporate the solvent. Reserpine, angiotensin II, melittin and insulin were used for the calibration. Differential Scanning Calorimetry (DSC) studies were carried out in a Q200 TA instrument. The DSC experiments were performed in three scans that included two heating scans (25 to 180 °C and −30 to 180 °C) and one cooling scan (180 to −30 °C) between them. The rate of heating/cooling was 10 °C min^−1^ under a nitrogen purge. The glass transition temperature (*T*_g_) is given as an inflection point, and the data presented are taken from the second heating scan. Polarized optical microscopy (POM). POM micrographs were obtained using a NIKON (MODEL) optical microscope, and photographs were taken using an iPhone 13. Entries 5 and 6 were mounted on glass slides as thin films melted at 130 °C using a hot plate with manual pressure applied between the two slides containing the sample and a cover glass, and then, an isotherm was performed at 94 °C overnight. The samples were cooled at room temperature before analysis. All samples were collected with a magnification of 40×.

### Computational methodology

We performed our calculations using density functional theory (DFT) with the Gaussian 09 package.^85^ All geometries were optimized in the gas phase with the ωB97X-D^86^ hybrid functional that includes Grimme's D2 empirical dispersion, which improves the description of non-covalent interactions, and the split-valence basis set of Ahlrichs and coworkers with double-ζ quality plus a set of polarization functions def2-SVPP,^87,88^ for all atoms. We used harmonic frequency calculations to characterize the stationary points that were found (geometries that are minima in energy only display positive frequencies, while structures that are maxima in energy possess only one negative frequency and are related to the transition state that describes the reaction coordinate). The thermal and entropy corrections to the total energy were computed at 298 K at 1 atm. To improve the approach of the electronic energy, we performed single-point calculations of the optimized geometries at the ωB97X-D/def2-TZVPP level.

## Results and discussion

### Ring-opening polymerization (ROP) of l-lactide (l-LA)

In the ROP of l-LA, the design of the polymerization conditions is very important, since its performance can be modulated through the modification of reaction parameters, such as the addition or exclusion of alcohols as initiators, the reaction time, the temperature, and the catalyst. There are some reports where the ROP of l-LA was carried out in the absence of an initiator, and rare-earth catalysts of samarium^33^ and lanthanum were used,^29^ leading to poor and null conversions, respectively. Besides the last compounds, catalysts such as those based on titanium,^36^ zinc,^21,25,35^ zirconium,^36^ hafnium,^36^ gallium^43^ and indium^43^ have been tested without the addition of any initiator. However, this species successfully achieved the ROP of l-LA.

Due to the findings mentioned in the last paragraph, we performed the catalytic study of p-block acetates with the elements in the fifth period, such as indium [In(iii)], tin [Sn(ii) and Sn(iv)] and antimony [Sb(iii)], in the ROP of l-LA without adding any initiator (entries 1–4, Table 2). In this series of trials, tin(ii) acetate displayed the best conversion with high polymerization degree (DP) (entry 2, Table 2), while the poorest results were obtained when indium(iii) acetate was used as the catalyst (entry 1, Table 2). Experimentally, the pattern of reactivity in terms of conversion toward ROP of l-LA is as follows: Sn(ii) > Sb(iii) > In(iii) ≅ Sn(iv). The soft metals (Pd^2+^, Cd^2+^, and Pt^2+^) have a lower charge. Conversely, hard metals carry a large charge (Ti^4+^, Fe^3+,^ and Co^3+^).^89^ These results suggest that the monomer l-LA prefers the softness of Sn(ii) *vs.* the hardness of Sn(iv).

**Table 2:** ROP of l-LA catalyzed by acetates from the p-block without adding the initiator^a^

Entry	Catalyst	Conv.^b^ (%)_NMR_	DP_NMR_^b^	*M*_n NMR_ c
1	[In(OAc)_3_]	29	6.3	450
2	[Sn(OAc)_2_]	80	47.7	3430
3	[Sn(OAc)_4_]	31	63.6	4580
4	[Sb(OAc)_3_]	70	11.0	790

a Reaction conditions: l-LA (20 mmol), catalyst (0.03 mmol), 140 °C, 80 min.

b The percentage of conversion and DP were obtained using ^1^H NMR spectroscopy in CDCl_3_.

c Calculated using the equation: *M*_n NMR_ = DP_NMR_ × MW_repetitive unit_ (72 g mol^−1^).

**Scheme 2:**
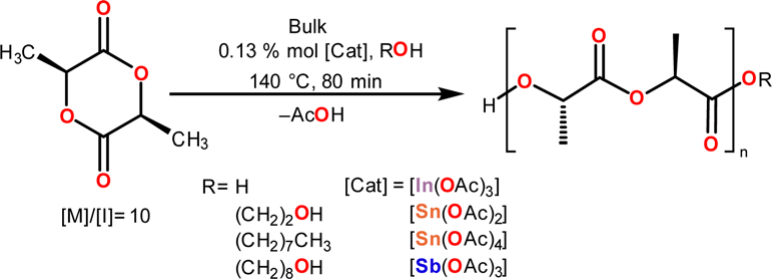
ROP of l-lactide (l-LA) catalyzed by In(iii), Sn(ii), Sn(iv) and Sb(iii) acetates using different initiators.

Previous studies have reported that impurities such as residual water (H_2_O) can act as an initiator.^90–94^ To evaluate our catalytic systems in moisture conditions, we tested all catalysts by adding water as an initiator (Scheme 2). The ROP results of l-lactide initiated by water are shown in Table 3, where we can appreciate an enhancement of the catalytic activity when indium and antimony acetates are used as catalysts (entries 5 and 8, Table 3).

**Table 3:** ROP of l-LA catalyzed by acetates from the p-block using water (H_2_O) as the initiator^a^

Entry	Catalyst	Conv.^b^ (%)_NMR_	DP_NMR_^b^	*M*_n NMR_ c	*T*_g_ (°C)
5^a^	[In(OAc)_3_]	54	6.1	440	−6
6^a^	[Sn(OAc)_2_]	66	17.0	1220	30, 41
7^a^	[Sn(OAc)_4_]	10	2.7	190	—
8^a^	[Sb(OAc)_3_]	95	9.1	660	24
8a^d^	[Sb(OAc)_3_]	100	7.4	530	13

a Reaction conditions: l-LA (20 mmol), H_2_O (2 mmol), catalyst (0.03 mmol), 140 °C, 80 min.

b The percentage of conversion and DP were obtained using ^1^H NMR spectroscopy in CDCl_3_.

c Calculated using the equation: *M*_n NMR_ = DP_NMR_ × MW_repetitive unit_ (72 g mol^−1^).

d Crude reaction product of entry 8 stored at 6 °C for one year.

**Fig. 1:**
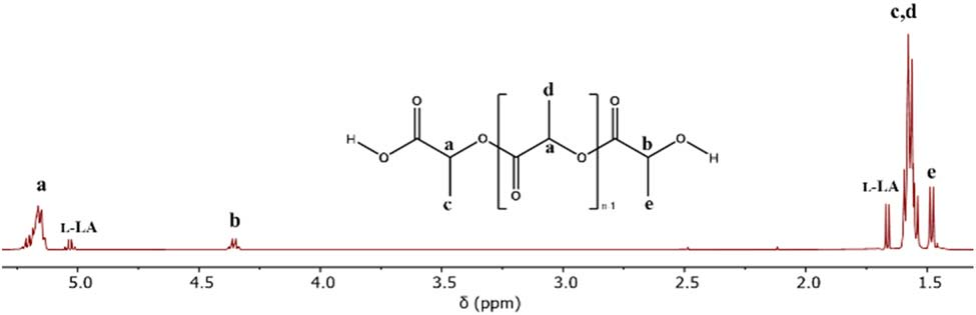
^1^H NMR spectrum of the oligomer obtained from the ROP of l-LA catalyzed by antimony(iii) acetate (entry 8, Table 3) in the presence of water as the initiator.

The theoretical product of the ROP of l-LA in the presence of water is the α-hydroxyl-ω-(carboxyl acid) PLLA (HOPLLACO_2_H). The first step to corroborating HOPLLACO_2_H as the product was the analysis of entry 8 catalyzed by Sb(iii) and using the ^1^H NMR spectrum (Fig. 1), where the triplet is attributed to methine attached to hydroxyl [b, –CH(CH_3_)–OH, *δ* 4.35]. Additionally, the methine of the repetitive unit [a, (–CO–CH(CH_3_)–O–)_*n*_, *δ* 5.16] was observed. Complementally, the ^13^C NMR spectrum confirmed the presence of the carboxyl acid end-group [1, –CO_2_H, *δ* 175.1] and a significant amount of the lactic acid dimer [1′,–CO_2_H, *δ* 175.3] (Fig. S4‡). The pattern in the carbonyl zone was similar to 6-hydroxicaproic acid, and their previously reported oligomers.^95^ The sample of entry 8 was stored at 6 °C for around one year (entry 8a, Table 3), and then analyzed by ^1^H NMR. Fig. S5B‡ illustrates a significant contrast with respect to the previous acquisition (Fig. 1). The main difference is the absence of the quadruplet attributed to the l-LA starting material. So, this evidence indicates that the reaction was in progress under 6 °C with a low rate constant, and suggests that Sb(iii) acetate is still active for a long period of time. A common reaction to demonstrate the presence of hydroxyl terminal groups is derivatization by trifluoroacetic anhydride (TFAA). In the case of entry 8a, after reaction with TFAA, the ^1^H NMR spectrum (Fig. S5A‡) showed a displacement of the quadruplet from 4.35 [b, –CH(CH_3_)–OH] to 5.34 ppm [b, –CH(CH_3_)–O–CO–CF_3_], evidencing the presence of trifluoroacetate end-groups. Consequently, the hydroxyl terminal groups' complementary mixed anhydride was also evidenced [f, a′, –CH(CH_3_)–CO–O–CO–CF_3_, *δ* 5.24], which is the product between carboxylic acid and TFAA. Conversely, the tin(ii) and tin(iv) acetates (entries 6 and 7, Table 3) exhibited decreased conversions with the addition of water (entries 2 and 3, Table 2).

**Fig. 2:**
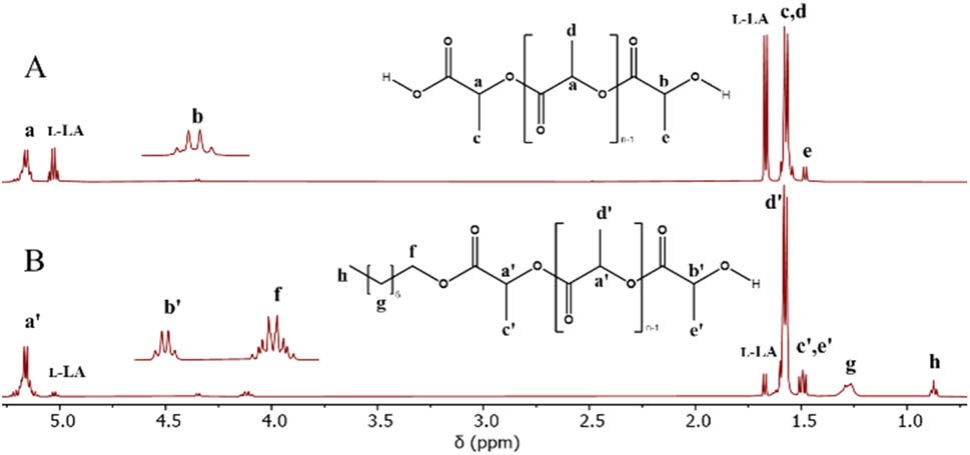
Comparative ^1^H NMR spectra of the oligomers obtained from ROP of l-LA catalyzed by tin(ii) in the presence of water (A, entry 6, Table 3) and 1-octanol (B, entry 10, Table 3) as initiators.

**Fig. 3:**
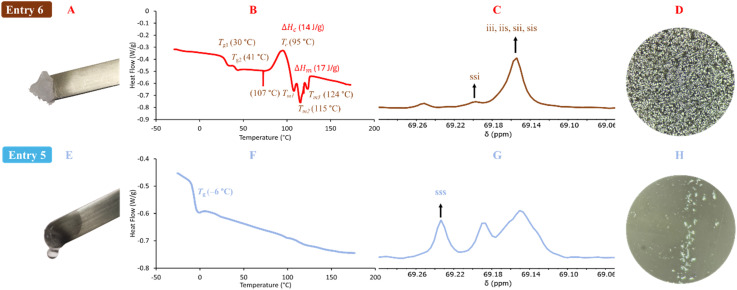
Products of the ROP of l-LA (Table 3) catalyzed by: indium(iii) acetate (bottom, entry 5) *vs.* tin(ii) acetate (top, entry 6). (A and E) macroscopic picture of the obtained oligomers; (B and F) DSC thermograms; (C and G) methine region of the ^13^C NMR spectrum (solvent: CDCl_3_, i = isotactic tetrad, s = syndiotactic tetrad); (D and H) POM micrographs (magnification: 40×).

The decrease in the activity of tin compounds may be due to the high concentration of water that promotes side reaction, such as the formation of hydroxides, stannoxane species, and tin oxides.^93,96–99^ The use of metal oxides in the ROP of lactones induces a heterogeneous system (solid–liquid), where the kinetics of polymerization becomes slow.^100,101^ The ^1^H NMR spectrum of the product of polymerization of l-LA catalyzed by tin(ii) (Fig. 2A) showed the same type of peaks with respect to Sb(iii) (Fig. 1) but with a significant amount of unreacted l-LA. It is evident that the oxidation states of tin, tin(ii) and tin(iv), result in a dramatic difference with respect to the conversion: 66% *vs.* 10%, respectively (Table 3). This gap between tin(ii) and tin(iv) remains both in the presence (Table 3) and absence (Table 2) of water as initiator. Only three systems in the presence of water as the initiator had a significant conversion that was in the upper 50%: In(iii), Sn(ii) and Sb(iii). However, Sn(ii) was the unique catalyst that produced a PLLA with the typical visual appearance of a semicrystalline oligomer (Fig. 3A), which was confirmed by DSC thermogram (Fig. 3B). Three types of transitions, such as glass transition temperature (*T*_g_ = 30, 41 °C), crystallization temperature (*T*_c_ = 95 °C) and melting temperature (*T*_m_ = 107, 115, 124 °C), were observed in the thermogram. These transitions for PLLA have been previously reported.^102,103^ Additionally, the pattern of tacticity for PLLA (entry 6) was confirmed by ^13^C NMR (Fig. 3C). Conversely, In(iii) and Sb(iii) exhibited the physical appearance of a polyester with an amorphous domain (Fig. 3E) and a characteristic *T*_g_ at −6 °C and 24 °C (Fig. 3F and S6B‡), respectively. The significant differences between In(iii) and Sb(iii) with respect to the *T*_g_ suggest the presence of a more flexible and amorphous oligoester when In(iii) (*T*_g_ = −6 °C) was used as the catalyst. To confirm the chemical nature of the oligoesters, ^13^C NMR analysis showed an atactic order in the methine carbons attributed to In(iii) (Fig. 3G). This evidence supports that under catalysis with In(III), the racemization of l-LA or PLLA can take place and poly(d,l-lactide) (PDLA) is eventually obtained. The ^13^C NMR spectrum of PDLA reported by previous authors^104,105^ has the same pattern of the oligoester catalyzed by In(iii) (Fig. 3G). In the case of Sb(iii), the degree of racemization (detected by ^13^C NMR) was lower than that for In(iii) (Fig. S7‡). This is consistent with a high value of *T*_g_ at 24 °C. All polymeric species shown in Table 3 are oligomers (from 440 to 1220 Da). Nevertheless, only the catalytic system of tin produces a semicrystalline oligoester, confirming that the selection of metal determines the type of product to obtain in terms of the physical properties. Additionally, it involves the molecular weight of PLLA not being the main parameter to induce an amorphous oligoester.

The physical properties of a couple of samples of oligomers obtained by Sn(ii) and In(iii) were visualized using polarized optical microscopy (POM) (Fig. 3D and H), where a series of spherulites are clearly observed for the PLLA sample from Sn(ii) catalysis (Fig. 3D). This evidence is consistent with that observed by DSC thermogram. Conversely, in the sample catalyzed by In(iii) (Fig. 3H), a blend of an amorphous domain attributed to PDLA and a small portion of crystalline domain of unreacted l-LA was seen. A study on the mechanism of racemization is currently underway in our laboratory, and it will be published in a future contribution.

In the scientific literature, it is well-known that metallic-alkoxides are strong bases and nucleophiles.^106,107^ We selected an aliphatic alcohol such as 1-octanol (C_8_H_17_OH) to produce *in situ* a new alkoxide using our previous acetate species. The requirement for using C_8_H_17_OH is its high boiling point (195 °C) and the miscibility with l-LA at high temperature (140 °C) under bulk conditions.^93,108–110^ The l-LA/C_8_H_17_OH molar ratio was equal to 10 to preserve the same proportion with respect to previous initiation with water (Table 3). In Table 4, the first four samples exhibited the results using C_8_H_17_OH as initiator (Scheme 2). The best conversion of the C_8_H_17_OH series (entries 9–12) is achieved by using tin(ii) (entry 10). Meanwhile, indium(iii) acetate (entry 9) displayed the lowest conversion. Comparing the chemical nature of Sn(ii) under different environments, the behavior of Sn(ii) as a nucleophilic agent was improved as an alkoxide (entry 10, conv. = 95%). On the contrary, in the presence of water, its nucleophilicity decreases (entry 6, Table 3, conv. = 66%).

**Table 4:** ROP of l-LA catalyzed by acetates from the p-block using alcohols and diols as initiators^a^

Entry	Catalyst	Initiator	Conv.^b^ (%)_NMR_	DP_NMR_^b^	*M*_n NMR_ c
9	[In(OAc)_3_]	1-Octanol	42	9.3	800
10	[Sn(OAc)_2_]	1-Octanol	95	19.4	1530
11	[Sn(OAc)_4_]	1-Octanol	75	14.9	1200
12	[Sb(OAc)_3_]	1-Octanol	50	10.7	900
13	[Sn(OAc)_2_]	Ethylene glycol	97	19.9	1490
14	[Sn(OAc)_2_]	1,8-Octanediol	96	19.2	1440

a Reaction conditions: l-LA (20 mmol), alcohol or diol (2 mmol), catalyst (0.03 mmol), 140 °C, 80 min.

b The percentage of conversion and DP were obtained using ^1^H NMR spectroscopy in CDCl_3_.

c Calculated using the equation: *M*_n NMR_ = DP_NMR_ × MW_repetitive unit_ (72 g mol^−1^) + MW_alcohol or diol_.

In polymer chemistry, the functionality called α,ω-hydroxy telechelic is important due to the synthesis of polyurethanes.^59,95,103,111–114^ In the ROP of l-LA, the ability to obtain an α,ω-hydroxy telechelic PLLA (HO-PLLA-OH) involves the use of a diol as the initiator.^59,103,115–118^ To diversify our oligomeric species, we explored two different aliphatic diols such as ethylene glycol (entry 13, Table 4) and 1,8-octanediol (entry 14, Table 4) as initiators in the presence of tin(ii) as the catalyst to synthesize HO-PLLA-OH (Scheme 2). The polymerization to obtain HO-PLLA-OH showed a similar high conversion (96–97%) with respect to PLLA-OH (entry 10, Table 4, conv. 95%). This suggests that both mechanisms of the reaction were initiated by tin(ii) alkoxides.

**Fig. 4:**
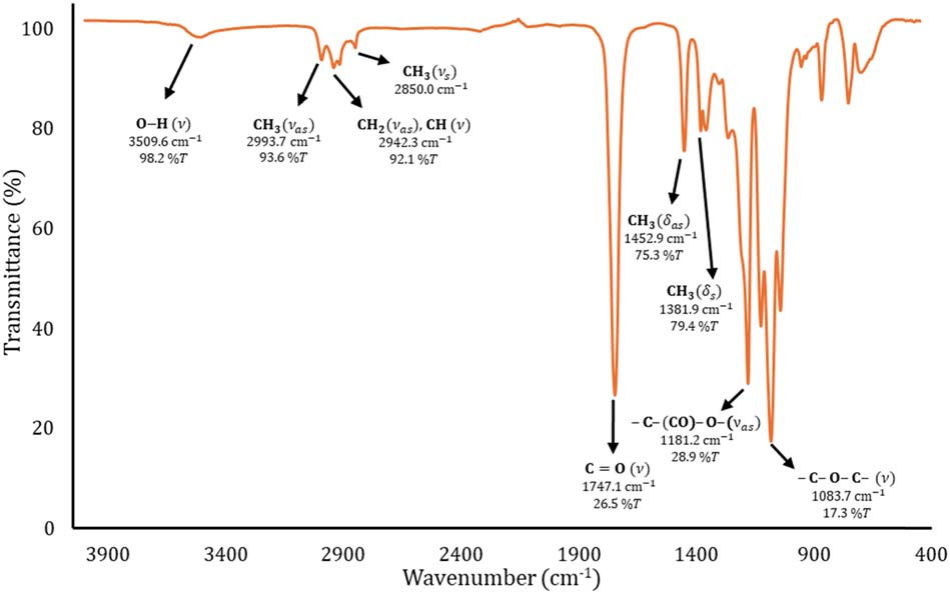
FT-IR spectrum of the oligomer obtained from the ROP of l-LA catalyzed by tin(ii) acetate in the presence of 1-octanol as the initiator (entry 10, Table 4).

To corroborate the terminal groups of PLLA-OH, in Fig. 2B, both end groups of α-hydroxyl-ω-methyl were detected at 4.3 (b′, –CH_2_–OH) and 0.8 ppm (h, –CH_3_), respectively. The complementary methylene of the octyl end-group was also observed at 4.1 ppm (f, –CH_2_–O–CO–). These findings are consistent with the analysis of the ^13^C NMR spectra shown in the ESI (Fig. S2).‡ In addition to the NMR data, the FT-IR spectra (Fig. 4) show the characteristic bands for hydroxyl (O–H, 3509 cm^−1^), methylene (CH_2_, 2993 cm^−1^) and carbonyl groups (C=O, 1747 cm^−1^) that help to confirm the presence of the ester repetitive unit and the hydroxyl end group. Another macromolecule (HO-PLLA-OH) was analyzed by ^1^H NMR (Fig. S8‡), where a couple of methine-attached hydroxyls [a, –CH(CH_3_)–OH] were detected at 4.35 ppm. The characterization and evidences of both samples (PLLA-OH and HO-PLLA-OH) confirmed that the system catalyzed by tin(ii) acetate is a versatile system that can use alcohols or diols in the preparation of α-hydroxyl-ω-methyl and α,ω-hydroxy telechelic polyesters. A complementary analysis of ^13^C NMR, as illustrated in Fig. S9,‡ suggests that the type of diol initiator can induce a small fraction of racemization, where ethylene glycol and 1,8-octanediol prevent and promote the racemization, respectively. A study of this effect is currently underway in our laboratory.

**Fig. 5:**
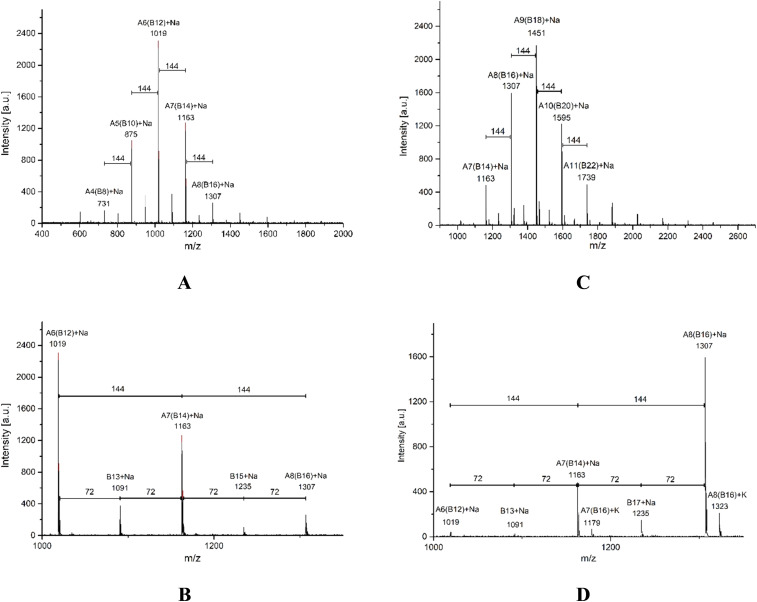
MALDI-TOF spectra of oligomers derived from ROP of l-LA using tin(ii) acetate and 1-octanol as the catalyst and initiator, respectively (entry 10, Table 4, crude product of the reaction [left (A and B)] *vs.* purified product by precipitation [right (C and D)]); the chemical structures are illustrated in Scheme 3.

**Scheme 3:**
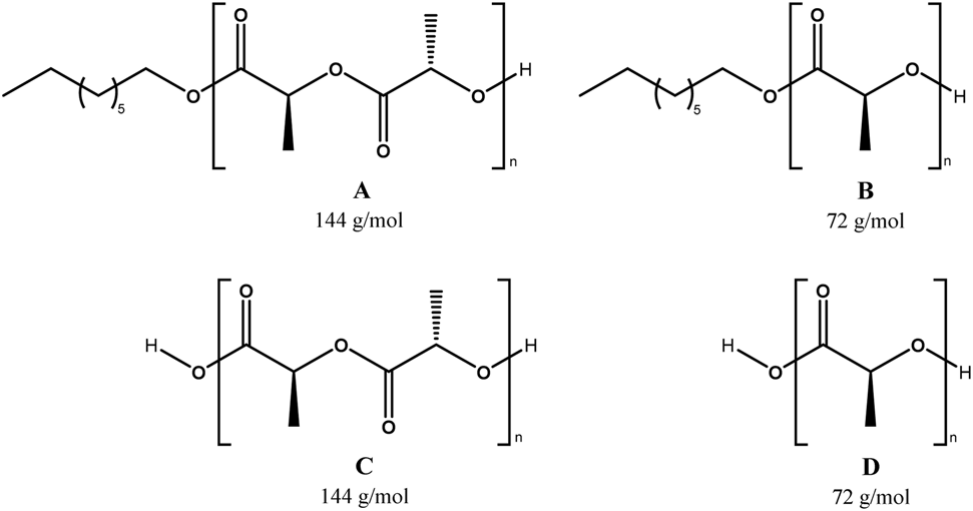
Species observed [PLLA obtained by ROP (A and C) and PLLA with the transesterification reaction (B and D)] in MALDI-TOF analysis, where the numbers indicate the molecular mass of the repetitive unit.

MALDI-TOF is a second characterization technique that can be used to corroborate the chemical nature of PLLA-OH previously detected by NMR. In Fig. 5A, the MALDI-TOF spectrum of crude PLLA-OH (entry 10, Table 4) is observed with a characteristic curve showing the molecular weight distribution of an oligoester, where the family of the main peaks are doped with sodium (Na^+^), forming the characteristic curve. The number-average molecular weight distribution (*M*_n_) calculated by MALDI-TOF was 1035 g mol^−1^ with a dispersity (*Đ*_M_) of 1.03. In previous contributions related to the synthesis of oligomers [poly(ε-caprolactone) (PCL)], the value of *M*_n_ calculated by NMR and MALDI-TOF was similar. In contrast, the *M*_n_ obtained by gel permeation chromatography (GPC) showed high values with respect to *M*_n NMR_ and *M*_n MALDI_. This effect was attributed to the polystyrene standards in the calibration curve of the GPC. Additionally, the *M*_n NMR_ is usually used in stochiometric calculations to produce poly(ester-urethanes) from PCL oligomers.^59,95,103,111–114^ The expanded view of Fig. 5A is illustrated in Fig. 5B, where fragments with DP from 6 to 8 are all doped with Na^+^. The gap between each peak is 144 g mol^−1^, which is consistent with the molecular weight of the monomer unit (l-LA) and represents the chemical structure A (Scheme 3). Complementarily, a new series of peaks with low intensity and different pattern in the sequence of the repetitive unit (72 g mol^−1^) was also observed (structure B, Scheme 3). This last pattern is a characteristic signal of an intermolecular transesterification during the propagation step. Some authors obtained similar results in previous studies.^119–124^ Additionally, the same repetitive unit of 72 g mol^−1^ was reported for the MALDI-TOF spectrum of poly(acid lactic) (PLA) using lactic acid as a monomer in a polycondensation reaction.^125–128^

A traditional method in the purification of polyesters is the precipitation,^16–21,23,25–27,29–38,40,53,59,60,62–64,66,67,129–148^ where the first solvent with a relatively low dielectric constant (4.8 for chloroform) is used to dissolve the polyester. Then, an excess of a second solvent with a high dielectric constant (32.7 for methanol) is added. The purpose of the precipitation is to isolate the product (polyester) from the unreacted monomer (lactone) by filtration, with the cost of some oligomers remaining in the mother liquor. The comparison between a crude product of the reaction and its precipitation has not been previously reported using MALDI-TOF. In this sense, the crude product of the reaction of PLLA-OH was previously discussed in the last paragraph. After precipitation with chloroform/methanol, the product was analyzed by MALDI-TOF, as seen in Fig. 5C. With respect to the crude product of the reaction (Fig. 5A), the representative peaks had a higher molecular weight, and *M*_n_ (MALDI-TOF) = 1468 g mol^−1^ with a dispersity (*Đ*_M_) of 1.03. The precipitation induces a displacement in the maximum peaks from 1019 (DP = 6, A6, Fig. 5A) to 1451 (DP = 9, A9, Fig. 5C). This is experimental evidence that the precipitation can move the curve of the molecular weight distribution to relatively high values (from left to right), as illustrated in Fig. 5B and D.

**Fig. 6:**
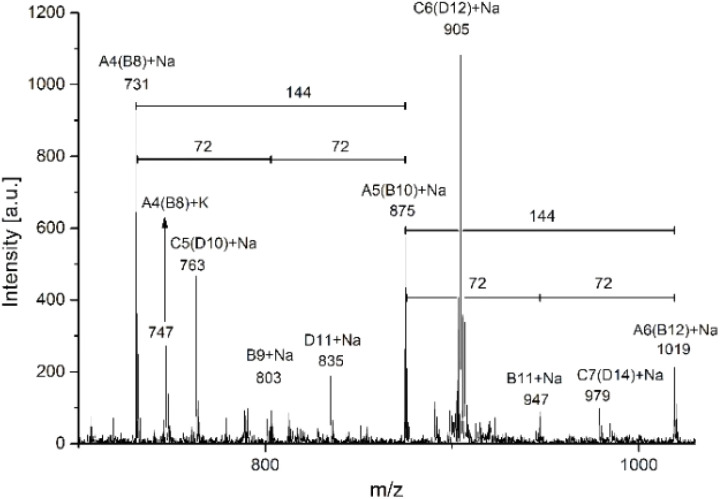
MALDI-TOF spectrum (expanded view) of the crude reaction of ROP of l-LA using 1-octanol as the initiator and indium(iii) acetate (B, entry 9, Table 4) as the catalyst.

Another catalytic system of interest was the ROP of l-LA catalyzed by indium(iii) acetate (InAc) and initiated by 1-octanol (entry 9, Table 4). In Fig. 6, the analysis by MALDI-TOF of the polyester obtained by the InAc catalyst exhibited similar types of peaks with respect to previous evidence with the system of tin(ii) acetate (Fig. 5B). Nevertheless, a new family of peaks attributed to a polyester with the α-hydroxyl-ω-carboxyl acid terminal groups (Scheme 3C and D) was detected. This result suggests that water (H_2_O) also acts as an initiator during depolymerization. This is because InAc is slightly more hygroscopic than tin(ii) acetate.

**Fig. 7:**
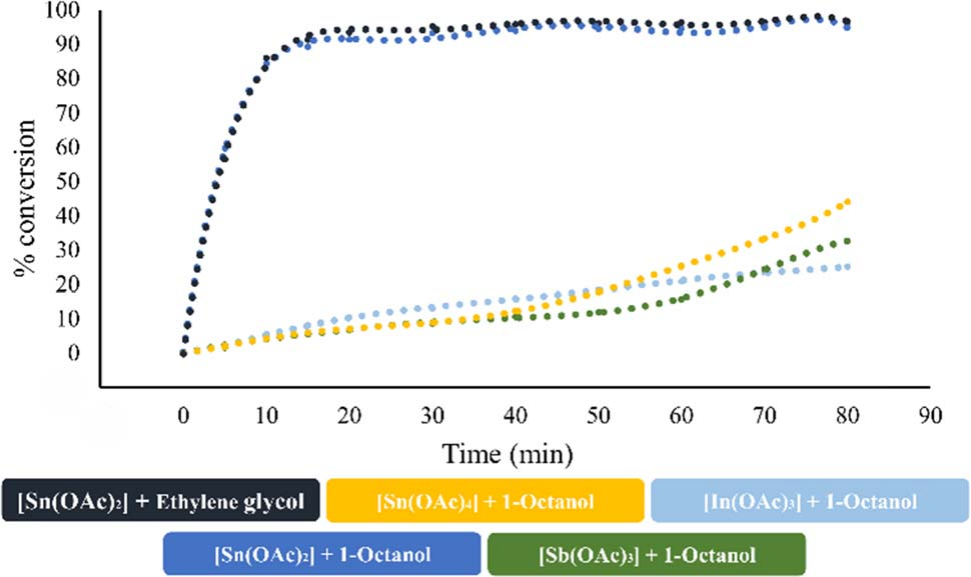
Effect of the three different elements [In(iii), Sn(ii), Sn(iv), and Sb(iii)] derived from acetates in the p-block in the catalysis of the ROP of l-LA at 140 °C.

**Fig. 8:**
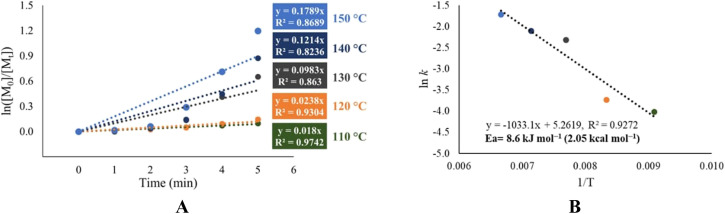
Kinetics of polymerization of l-LA catalyzed by tin(ii) acetate (1-octanol as initiator) at different temperatures (110–150 °C) to obtain constant rates (*k*) using a semilogarithmic plot (A), and the quantification of the activation energy using the Arrhenius equation: plot of ln *k vs.* 1/*T* (B).

Due to the interesting catalytic behavior variation (entries 9–12, Table 4) in the ROP of l-LA using 1-octanol as an initiator, we focused our efforts to analyze the chemical kinetics of these polymerization systems. The results obtained from these studies are shown in Fig. 7. In these data, we can observe the catalytic efficiency of the tin(ii) acetate catalyst since it achieves a conversion of 90% in the first 20 minutes of reaction, even when using ethylene glycol as the initiator. Comparing the system of tin(ii) acetate *vs.* the rest of the catalysts [In(iii) acetate, tin(iv) acetate and antimony(iii)acetate], tin(ii) acetate showed a gap in terms of the conversion of 80% over the rest of the catalysts at 20 minutes. Due to the observed fast conversion in a short period of time in the ROP catalyzed by tin(iii) acetate, we focused on the first five minutes of the polymerization, taking aliquots for every minute of the trials carried out at temperatures of 110, 120, 130, 140 and 150 °C (Fig. S10‡). The results of the kinetics study (pseudo first-order reaction) by varying temperatures are shown in Fig. 8A, where the conversion after five minutes of polymerization reaction congruently falls from the highest to the lowest temperature condition. This is evidenced by the obtained rate constant (*k*) of each kinetic step, showing that *k* is proportional to the temperature. In Fig. 8B, we show the semilogarithmic plot according to the Arrhenius equation of the ROP of l-lactide, which displays a linear pattern, involving an activation energy (*E*_a_) of 8.6 kJ mol^−1^ (2.05 kcal mol^−1^). Comparing our result with respect to a previous publication using bismuth(iii) subsalicylate,^59^ tin(ii) acetate has a significantly high activity with respect to bismuth(iii) acetate.

**Fig. 9:**
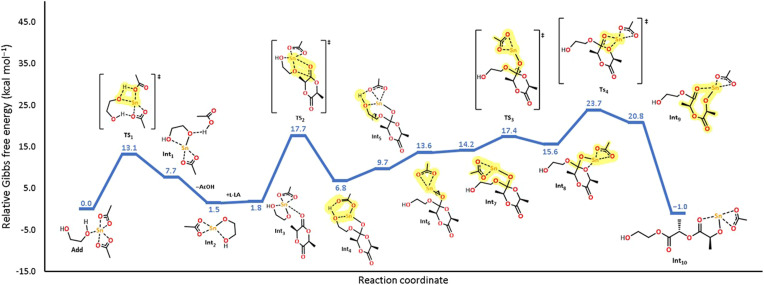
Energy profile of the initiation step of the ROP mechanism of l-LA initiated by tin acetate and ethylene glycol as the catalyst and initiator, respectively, at room temperature.

In Fig. 9, we show the mechanistic study using density functional theory (DFT) of the initiation step for the ROP of l-LA using tin(ii) acetate and ethylene glycol as the catalyst and initiator, respectively. The selection of ethylene glycol (EG) was due to (1) optimizing the computational cost, (2) the similar reactivity with respect to 1-octanol (Fig. 7), and (3) the use of EG as an initiator in a mechanism reaction of ROP catalyzed by bismuth(iii) acetate.^59^ In this energy profile, the generation of the tin alkoxide (Int_1_) proceeds through the proton transfer from ethylene glycol to the carboxylate ligand with an energetic cost of 13.1 kcal mol^−1^ (TS_1_). In the next step, the tin atom in the alkoxide is coordinated by the carbonylic oxygen in l-LA to form the coordination adduct (Int_3_) and thus carry out the nucleophilic attack (TS_2_), which displays an energy barrier of 17.7 kcal mol^−1^. Once the nucleophilic attack occurred, the generated intermediate undergoes small structural arrangements to promote the C–O bond rotation (TS_3_, 17.4 kcal mol^−1^) that places the—Sn(OAc) moiety in parallel to the endocyclic oxygen of the lactone (Int_8_). The importance of this step is to promote an effective O–Sn interaction that favors the ring-opening of l-LA (TS_4_), which is the rate-determining step (23.7 kcal mol^−1^). It is important to mention that the proposed coordination-insertion mechanism for tin(ii) acetate in this study displays a similar pattern to the one reported for bismuth(iii) subsalicylate,^59^ but with differences in the values of the energy barriers. Computational chemistry assumes a single monomer molecule during the initiation step in the polymerization reaction. The calculation was performed in the gas-phase in a vacuum. However, in reality, the conditions of our experiments in the laboratory are different in terms of moles (Avogadro constant), bulk polymerization, interactions between two or more molecules of l-LA or the metal catalyst, collision theory, molecular mechanics, *etc.* So, the expectation to have a match between the computational and experimental results is non-trivial. Consequently, both results (computational and experimental) only demonstrated the feasibility of the reactions.

Finally, we have a wide perspective of the catalysts reported in this work. A series of different catalytic systems are described in Table 5. The main parameter to compare between them is the % mol of catalyst utilized in each reaction. It is remarkable that the system reported in this work (In, Sn and Sb) presented a low value with respect to the rest of the metals. Additionally, our system works under solvent-free conditions (bulk polymerization), showing from moderate to excellent conversions in the absence of an inert atmosphere and short reaction times. According to the analyzed results in this article, these ones represent clear evidence on the importance of choosing or designing the correct catalyst for the ROP of lactides that allows access to PLLA materials with the desired crystallinity, giving the design of poly(lactides) with the optimal physical properties that can satisfy the needs of the targeted application.

**Table 5:** Catalytic conditions in different assisted chemical reactions

Catalysts	Reaction	Solvent	Temp. (°C)	% mol	% conv.	*t* (min)	Atm.
In, Sn, Sb (this work)	ROP l-lactide	Free	140	0.13	40–96	80	Air
Ti^39^	ROP l-lactide	Toluene	60	0.95	99	220	Air
Zn^51^	ROP l-lactide	Free	150	0.2	96	4	Argon
Zr^40^	ROP l-lactide	Benzene	90	0.99	83	270	Nitrogen
Al^56^	ROP l-lactide	Toluene	90	0.50	92	1500	Nitrogen
Ir^149^	C–H activation	PhCl	135	10	78	1440	Argon
Ga^150^	Hydroarylation	DCE	80	5	93	240	Argon
In^151^	Retro-Claisen condensation	Free	80	5	97	1440	Argon
Sn^152^	Esterification reactions	CH_3_CN	25	10	89	360	Air
Sb^153^	One-pot synthesis	Free	25	2.2	96	1	Air

## Conclusions

Four different catalytic systems derived from metal-acetates, such as indium [In(iii)], tin [Sn(ii) and Sn(iv)] and antimony [Sb(iii)], were studied in the ring-opening polymerization (ROP) of l-lactide (l-LA) in the absence and presence of initiators (1-octanol, ethylene glycol, 1,8-octanediol, and water). Under the addition of 1-octanol as an initiator, In(iii), Sn(iv) and Sb(iii) exhibited the lowest performance with a poor conversion value. However, Sn(ii) had the highest reactivity with excellent conversion. The poly(l-lactide) (PLLA) obtained by tin(ii) acetate preserves its stereochemistry, obtaining a PLLA semicrystalline structure. Conversely, the oligoesters prepared by In(iii) acetate showed racemization in the main chain. Consequently, a sample with an amorphous domain was detected. A light transesterification was detected by MALDI-TOF. The reaction mechanism was validated using combined experimental and computational chemistry approaches, where a transfer reaction forming a tin alkoxide precedes the nucleophilic attack to l-LA. Then, the ring-opening of l-LA, which is identified as the rate-determining step, takes pace. The catalytic system of tin(ii) acetate studied in this work represents an alternative route to obtain PLLA, and its comparison with In(iii), Sn(iv) and Sb(iii) acetates brings a better perspective to the ROP of lactones. The choice of an element as a catalyst in the ROP of l-LA is a non-trivial factor because the physical properties of the PLLA product can change from an amorphous form to a semicrystalline polyester.

## Data availability

The data supporting this article have been included as part of the ESI.‡

## Author contributions

Oscar F. González-Belman: investigation, validation, formal analysis, writing – original draft. J. Oscar C. Jiménez-Halla: supervision. Gerardo González: supervision. José E. Báez: conceptualization, formal analysis, supervision, writing – original draft, writing – review, funding acquisition.

## Conflicts of interest

There are no conflicts to declare.

## Supplementary Material

RA-014-D4RA06783E-s001
