# Supplementary material for: Comparison of three elements (In, Sn, and Sb) in the same period as catalysts in the ring-opening polymerization of l-lactide: from amorphous to semicrystalline polyesters
Source: RSC Adv. 2024 Oct 31;14(47):34733–45. doi: 10.1039/d4ra06783e (PMC11526846; doi:10.1039/d4ra06783e)
Supplement: RA-014-D4RA06783E-s001 [file RA-014-D4RA06783E-s001.pdf]

Supplementary Information (SI) for RSC Advances.
This journal is © The Royal Society of Chemistry 2024

Supplementary Information

Comparison of three different elements in the same period (In, Sn, and Sb) as catalysts in the ring-opening polymerization of L-lactide: from amorphous to semicrystalline polyester

Oscar F. González-Belman, J. Oscar C. Jiménez-Halla, Gerardo González, and José E. Báez *[a]

Department of Chemistry, Division of Natural and Exact Sciences, University of Guanajuato (UG), Noria Alta S/N 36050, Guanajuato, Mexico.

To whom correspondence should be addressed: José E. Báez, e-mail: jebaez@ugto.mx

Table of Contents

Figures		Page
	**Figure S1.** ^{13}C NMR spectrum of the oligomers obtained from ROP of L-lactide catalyzed by tin (III) acetate (Entry 10, Table 4) in presence of 1-octanol as initiator.	**4**
	**Figure S2.** ^{13}C NMR spectrum of the oligomers obtained from ROP of L-lactide catalyzed by tin (III) acetate (Entry 10, Table 4) in presence of 1-octanol as initiator.	**4**
	**Figure S3.** Thermograms (DSC) of oligomers obtained from ROP of L-LA initiated by $Sn(OAc)_2$ and 1-octanol: A) Purified oligomers and B) Reaction crude.	**5**
	**Figure S4.** ^{13}C NMR spectrum of the carbonyl region of the oligomers obtained from the ROP of L-LA catalyzed by antimony(III) acetate (Entry 8, Table 3) in presence of water (H_2O) as initiator.	**5**
	**Figure S5.** ^{1}H NMR spectrum of methine region of oligomers obtained from ROP L-LA catalyzed by antimony(III) acetate (Entry 8, Table 3) in presence of water (H_2O) as initiator: A) After the derivatization reaction and B) before the derivatization reaction.	**6**
	**Figure S6.** Thermograms (DSC) of oligomers obtained from ROP of L-LA using water (H_2O) as initiator and tin(II) acetate (A, Entry 6, Table 3) and antimony(III) acetate (B, Entry 8, Table 3) as catalysts.	**6**
	**Figure S7.** ^{1}H NMR spectrum of the oligomers obtained from ROP of L-lactide catalyzed by tin(II) acetate in presence of ethylene glycol (A, Entry 13, Table 4) and 1,8-octanediol (B, Entry 14, Table 4) as initiators.	**7**
	**Figure S8.** ^{13}C NMR spectrum of methine [-OOC-CH(CH_3)-O-] region of the oligomers obtained from ROP of L-LA catalyzed by tin(II) acetate in presence of 1,8-octanediol (A) and ethylene glycol (B) as initiators (Solvent: $CDCl_3$, i = isotactic diad, s = syndiotactic diad).	**7**
	**Figure S9.** Thermal effect in the ROP of L-lactide initiated by tin(II) acetate and 1-octanol.	**8**
	**Figure S10.** Thermal effect in the ROP of L-lactide initiated by tin(II) acetate and 1-octanol.	**8**
	**Figure S11.** Thermograms (DSC) of oligomers obtained from ROP of L-LA using water (H_2O) as initiator and antimony(III) acetate (B, Entry 8, Table 3) as catalyst: after the reaction time finishes (B) and one year later (A).	**9**

Tables

Table S1. Optimized cartesian coordinates (xyz) for all species in the energy profile of Figure 9. Coordinates are given in Å. **9**

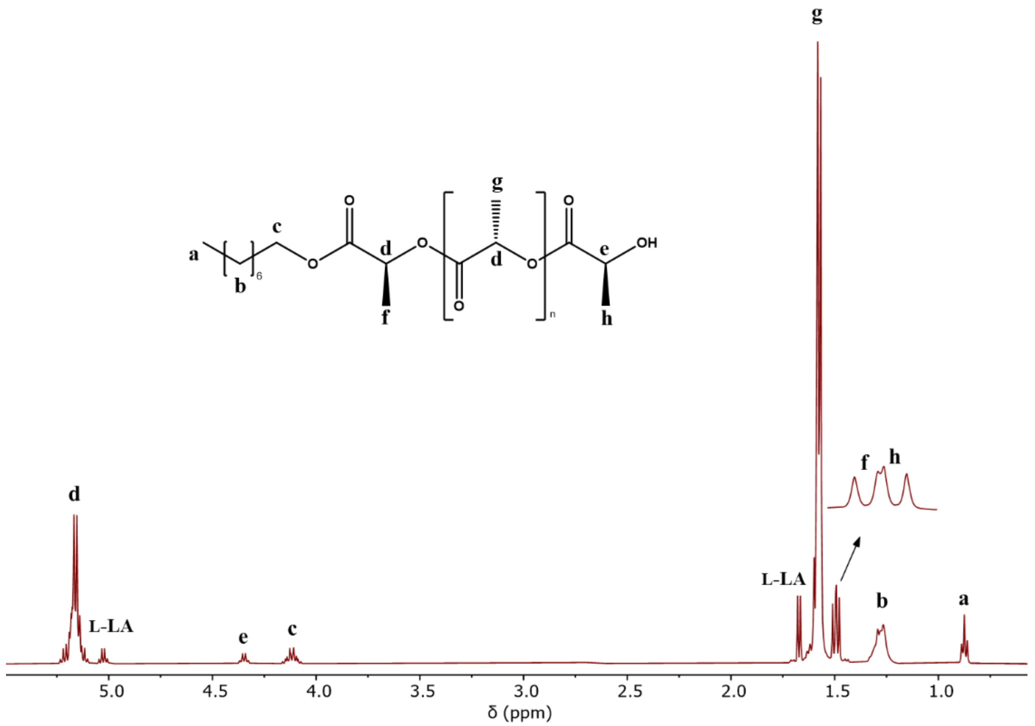

Figure S1. ^{1}H NMR spectrum of the oligomers obtained from ROP of L-lactide catalyzed by tin(III) acetate (Entry 10, Table 4) in presence of 1-octanol as initiator.

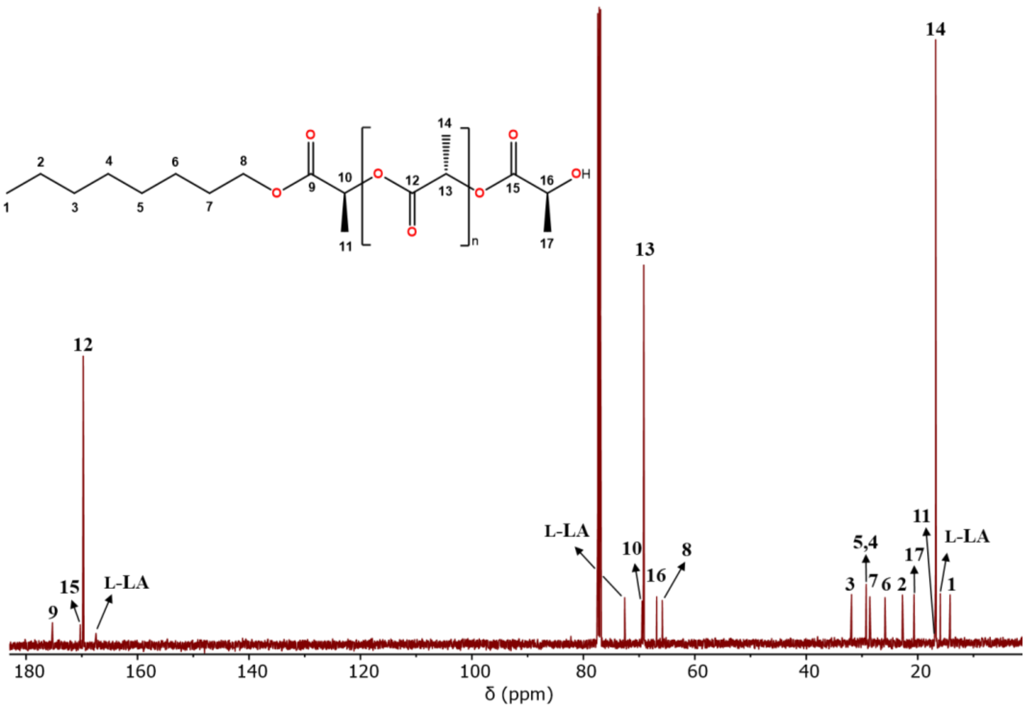

Figure S2. ^{13}C NMR spectrum of the oligomers obtained from ROP of L-lactide catalyzed by tin (III) acetate (Entry 10, Table 4) in presence of 1-octanol as initiator.

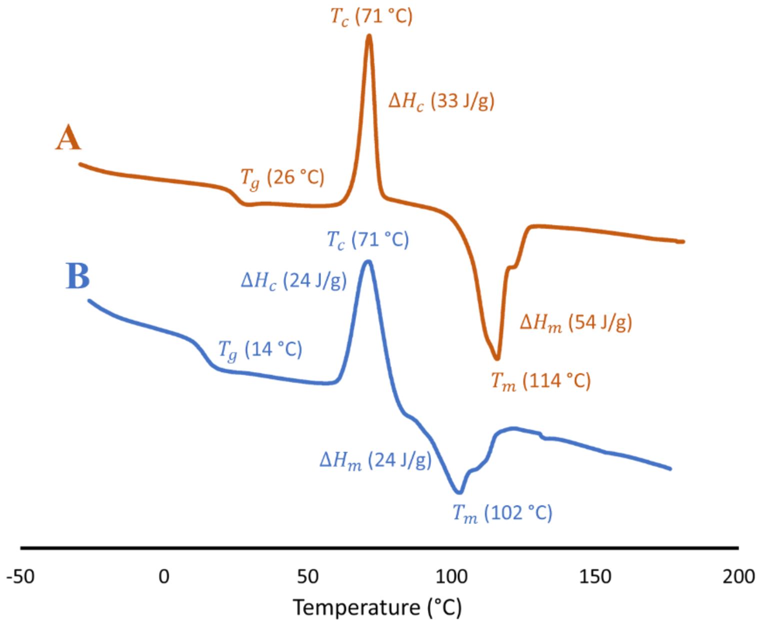

Figure S3. Thermograms (DSC) of oligomers obtained from ROP of L-LA initiated by $Sn(OAc)_2$ and 1-octanol: A) Purified oligomers and B) Reaction crude.

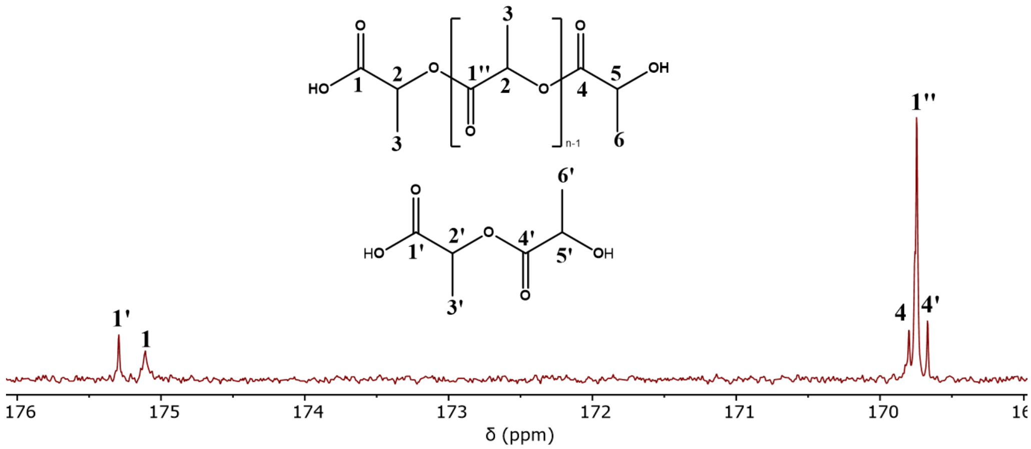

Figure S4. ^{13}C NMR spectrum of the carbonyl region of the oligomers obtained from the ROP of L-LA catalyzed by antimony(III) acetate (Entry 8, Table 3) in presence of water (H_2O) as initiator.

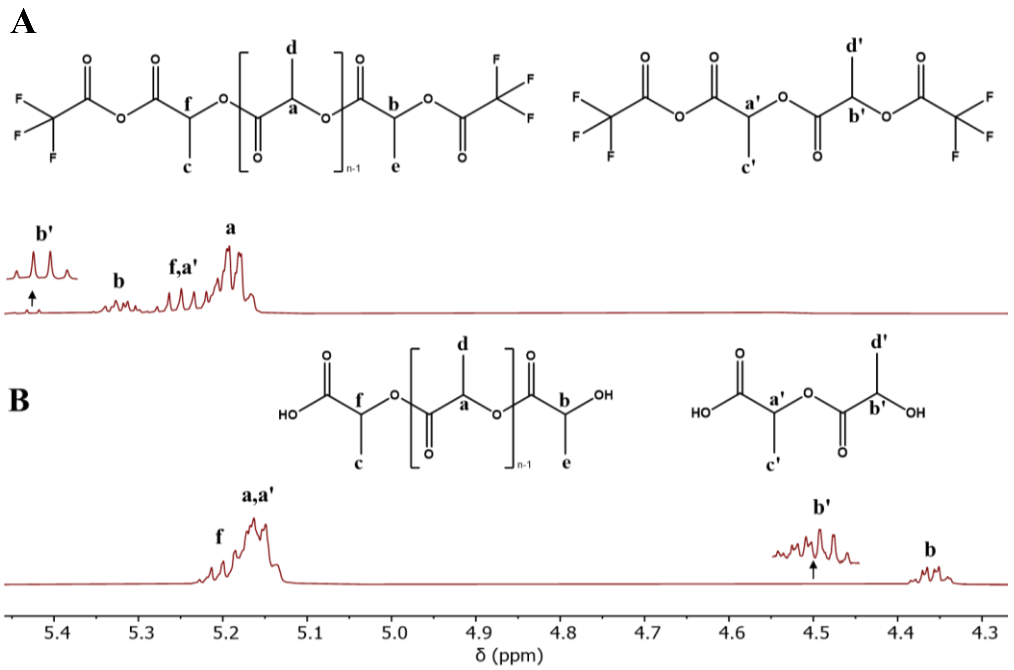

Figure S5. ^{1}H NMR spectrum of methine region of oligomers obtained from ROP L-LA catalyzed by antimony(III) acetate (Entry 8, Table 3) in presence of water (H_2O) as initiator: A) After the derivatization reaction and B) before the derivatization reaction.

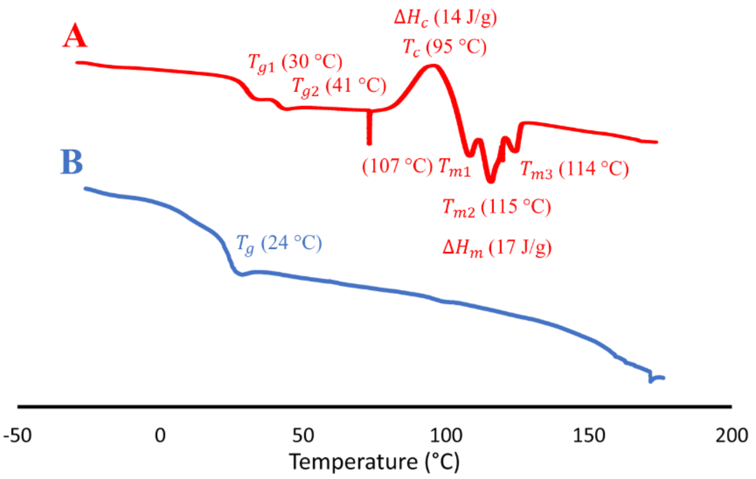

Figure S6. Thermograms (DSC) of oligomers obtained from ROP of L-LA using water (H_2O) as initiator and tin(II) acetate (A, Entry 6, Table 3) and antimony(III) acetate (B, Entry 8, Table 3) as catalysts.

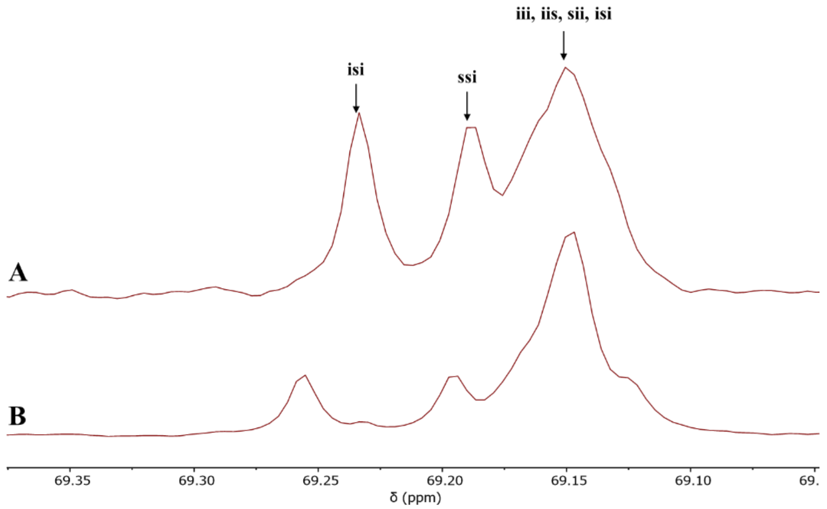

Figure S7. ^{13}C NMR spectrum of methine [-OOC-CH(CH_3)-O-] region of the oligomers obtained from ROP of L-LA catalyzed by indium(II) (A) acetate and antimony(III) acetate (B) in presence of water (H_2O) as initiator (Solvent: $CDCl_3$, i = isotactic tetrad, s = syndiotactic tetrad).

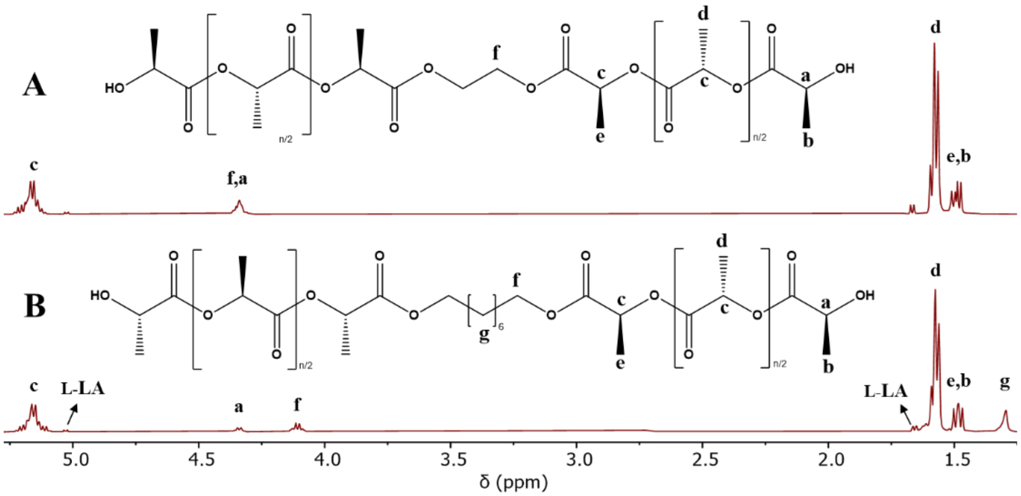

Figure S8. ^{1}H NMR spectrum of the oligomers obtained from ROP of L-lactide catalyzed by tin(II) acetate in presence of ethylene glycol (A, Entry 13, Table 4) and 1,8-octanediol (B, Entry 14, Table 4) as initiators.

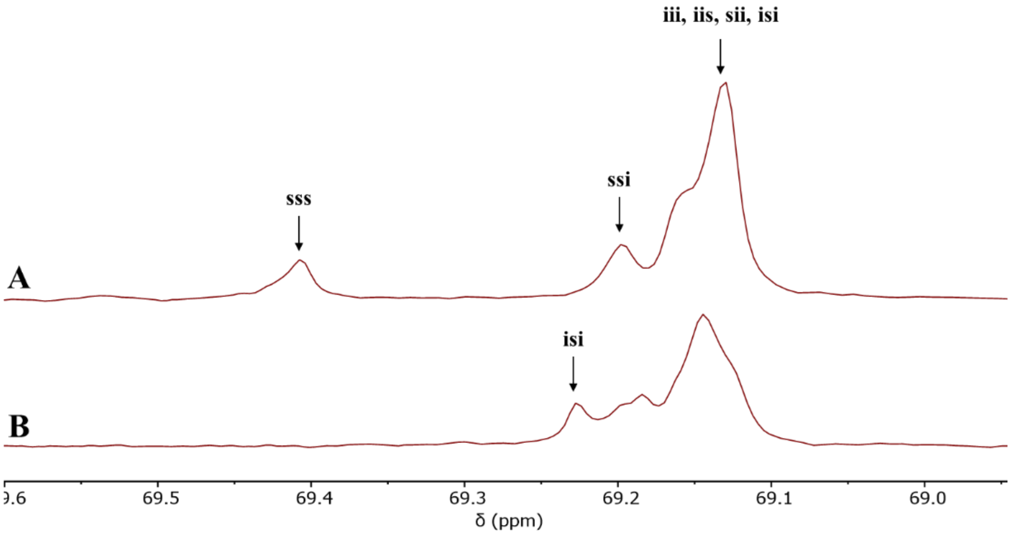

Figure S9. ^{13}C NMR spectrum of methine [-OOC-CH(CH_3)-O-] region of the oligomers obtained from ROP of L-LA catalyzed by tin(II) acetate in presence of 1,8-octanediol (A) and ethylene glycol (B) as initiators (Solvent: $CDCl_3$, i = isotactic tetrad, s = syndiotactic tetrad).

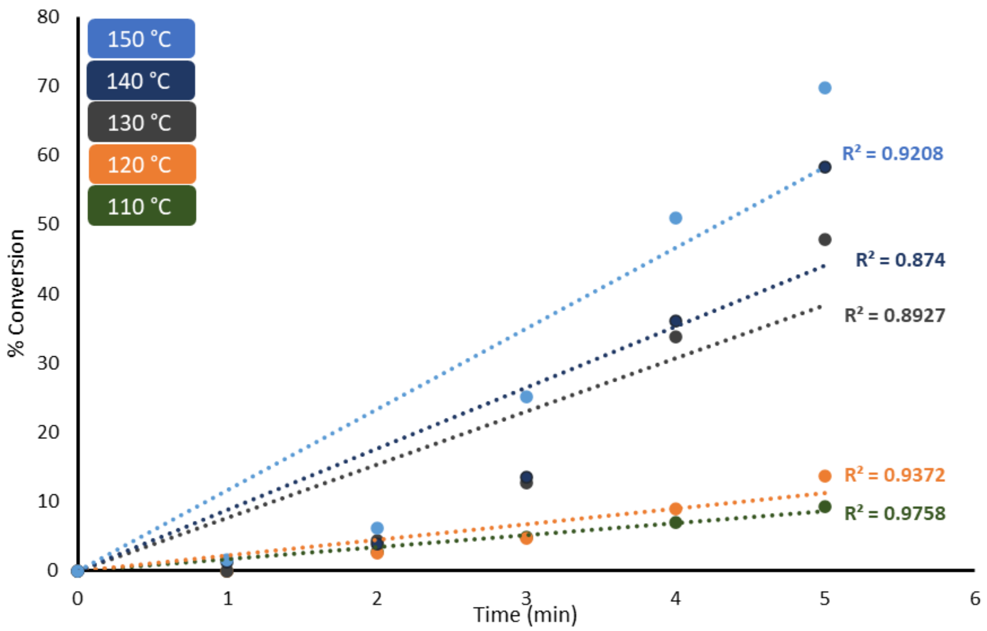

Figure S10. Thermal effect in the ROP of L-lactide initiated by tin(II) acetate and 1-octanol.

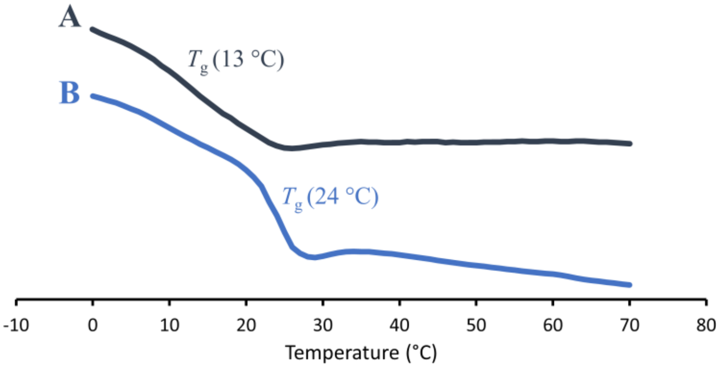

Figure S11. Thermograms (DSC) of oligomers obtained from ROP of L-LA using water (H_2O) as initiator and antimony(III) acetate (B, Entry 8, Table 3) as catalyst, respectively: after the reaction time finishes (B) and one year later (A).

Table S1. Optimized cartesian coordinates (xyz) for all species in the energy profile of Figure 9. Coordinates are given in Å.

Add
E(scf)= –900.931340804

50	0.707313000	0.083547000	-1.072647000
8	1.716436000	1.421843000	0.519895000
8	0.166398000	-1.179090000	0.637000000
6	0.665748000	2.091699000	0.702244000
6	1.284348000	-1.798257000	0.811943000
6	1.364872000	-2.750084000	1.977289000
8	2.236428000	-1.600616000	0.050855000
6	0.625734000	3.182954000	1.736098000
8	-0.369490000	1.823271000	0.028789000
1	0.203853000	2.761744000	2.664955000
1	-0.034707000	3.997830000	1.404511000
1	1.638341000	3.555831000	1.945873000
1	2.332875000	-3.269650000	1.983191000
1	0.540203000	-3.479338000	1.923572000
1	1.242118000	-2.181754000	2.914294000
8	-1.852133000	-0.066370000	-1.158727000
6	-2.645019000	-1.123914000	-0.658905000
1	-2.077118000	-2.062542000	-0.758952000
1	-3.566630000	-1.211021000	-1.270463000
1	-2.071483000	0.736127000	-0.643546000
6	-2.995650000	-0.862701000	0.797569000
1	-3.672867000	-1.652345000	1.178687000
1	-2.070179000	-0.873216000	1.391542000
8	-3.547932000	0.428285000	0.942274000
1	-4.427904000	0.444202000	0.540596000

TS_1
E(scf)= –900.913850235
ν_{min}= –726.4915

50	0.207299000	-0.181284000	-1.032709000
8	-1.750995000	-1.076018000	-0.024134000
8	1.716568000	1.527489000	-0.095116000
6	-2.895869000	-0.416677000	0.181225000
6	0.689724000	2.109690000	0.326275000
6	0.782716000	3.362872000	1.151902000
8	-0.460723000	1.630534000	0.074993000
6	-3.286563000	-0.318932000	1.640162000
8	-3.529795000	0.054214000	-0.724113000
1	-3.339253000	-1.322409000	2.095205000
1	-4.257070000	0.187255000	1.738744000
1	-2.512748000	0.256424000	2.177616000
1	0.602240000	3.099323000	2.208251000
1	-0.000431000	4.073925000	0.848159000
1	1.779392000	3.816557000	1.059125000
8	0.297240000	-1.027233000	1.006453000
6	1.220791000	-2.035384000	1.312033000
1	1.079610000	-2.925590000	0.665070000
1	1.093010000	-2.358067000	2.361515000
1	-0.872564000	-1.225552000	0.799796000
6	2.621712000	-1.476205000	1.117993000
1	2.790112000	-0.671723000	1.855586000
1	3.373346000	-2.267756000	1.287778000
8	2.754626000	-0.976039000	-0.198456000
1	2.888995000	-0.013957000	-0.155963000

Int_1
E(scf)= –900.923375124

50	0.526518000	-0.259586000	-1.011223000
8	-2.026124000	-1.343637000	-0.256448000
8	1.452157000	1.678610000	-0.045599000

Int2
E(scf)= –672.075170019

50	0.210312000	-0.966887000	-0.212646000
8	-1.168909000	0.804618000	-0.791115000
6	-2.026973000	0.475800000	0.085355000

6	-3.055965000	-0.572875000	0.156970000
6	0.297185000	2.132517000	0.191216000
6	0.121148000	3.445602000	0.901968000
8	-0.716631000	1.463223000	-0.148710000
6	-2.983414000	-0.168672000	1.613666000
8	-3.933636000	-0.270280000	-0.596960000
1	-3.011162000	-1.063507000	2.259946000
1	-3.833168000	0.484305000	1.855682000
1	-2.034256000	0.359420000	1.800599000
1	-0.047590000	3.240854000	1.973532000
1	-0.766092000	3.968637000	0.515631000
1	1.021255000	4.068230000	0.799482000
8	0.247783000	-1.154160000	0.856530000
6	1.296099000	-1.846095000	1.452228000
1	1.499703000	-2.813455000	0.943812000
1	1.046838000	-2.080011000	2.505115000
1	-1.340227000	-1.480136000	0.451887000
6	2.561129000	-1.003239000	1.431544000
1	2.397106000	-0.086906000	2.024817000
1	3.420237000	-1.559563000	1.847145000
8	2.822624000	-0.654387000	0.073495000
1	3.134999000	0.263052000	0.039498000

Int_3

E(scf)= −1205.89118786

8	-2.420869000	0.137993000	-1.218917000
6	-1.550626000	-0.806418000	-0.897084000
6	-1.723080000	-1.443129000	0.473527000
8	-0.671535000	-1.122294000	-1.663305000
8	-3.080334000	-1.374541000	0.921141000
6	-3.132955000	0.780635000	-0.155162000
6	-3.795458000	-0.260461000	0.739216000
8	-4.867258000	-0.115727000	1.249486000
6	-4.121088000	1.748852000	-0.763060000
1	-2.380148000	1.322899000	0.445951000
1	-4.855588000	1.212685000	-1.384627000
1	-3.584673000	2.480591000	-1.386943000
1	-4.661333000	2.274721000	0.038042000
6	-1.295458000	-2.895208000	0.495953000
1	-1.077345000	-0.862580000	1.158396000
1	-1.880494000	-3.487620000	-0.225799000
1	-1.456612000	-3.301765000	1.506562000
1	-0.226007000	-2.947076000	0.250508000
50	1.675737000	0.057753000	-0.986026000
8	2.017652000	1.942271000	0.475986000
6	0.779949000	2.061806000	0.644832000
6	0.224257000	3.099053000	1.585529000
8	-0.021207000	1.293609000	0.035963000
1	-0.166517000	2.590241000	2.483395000
1	-0.616668000	3.627119000	1.108787000
1	1.002688000	3.814098000	1.886033000
8	1.426036000	-1.191208000	0.627181000
6	2.540627000	-1.764638000	1.220404000
1	3.021024000	-2.528244000	0.568205000
1	2.246777000	-2.285622000	2.153205000
6	3.577929000	-0.705672000	1.567083000
1	3.148885000	-0.005394000	2.305148000
1	4.491338000	-1.161965000	1.989702000
8	3.902128000	-0.003452000	0.370110000

6	-3.274296000	1.301791000	0.245062000
8	-1.823589000	-0.527339000	0.811680000
1	-2.996398000	2.291022000	0.646076000
1	-3.978107000	0.813562000	0.933046000
1	-3.743195000	1.462194000	-0.738709000
8	1.104418000	0.011207000	1.329341000
6	2.213752000	0.792678000	1.055207000
1	3.111022000	0.187496000	0.790333000
1	2.488651000	1.390055000	1.947099000
6	1.925890000	1.752242000	-0.093293000
1	1.118365000	2.443206000	0.204247000
1	2.822737000	2.333555000	-0.372328000
8	1.498956000	0.971307000	-1.212000000
1	0.744083000	1.409824000	-1.634711000

TS_2

E(scf)= −1205.87908918

ν_{min}= −209.2538

8	-1.034666000	0.585767000	-0.114866000
6	-0.948914000	-0.731523000	-0.414216000
6	-2.249479000	-1.498705000	-0.146942000
8	-0.183380000	-1.031833000	-1.363726000
8	-3.360352000	-0.683060000	-0.536960000
6	-2.151620000	1.044273000	0.637125000
6	-3.435845000	0.560865000	-0.042484000
8	-4.425926000	1.220279000	-0.151380000
6	-2.063350000	2.551897000	0.725509000
1	-2.116333000	0.605950000	1.654243000
1	-2.162763000	2.998212000	-0.276364000
1	-1.087356000	2.840795000	1.147995000
1	-2.874414000	2.940197000	1.359303000
6	-2.318223000	-2.801064000	-0.905152000
1	-2.330790000	-1.685335000	0.937035000
1	-2.213658000	-2.617735000	-1.985138000
1	-3.280706000	-3.298101000	-0.707087000
1	-1.495402000	-3.454230000	-0.577279000
50	1.878603000	-0.988844000	-0.406505000
8	1.719623000	2.482808000	0.541082000
6	1.597639000	2.193734000	-0.658637000
6	1.110333000	3.223762000	-1.646082000
8	1.854235000	1.038938000	-1.153139000
1	0.120815000	2.902367000	-2.013564000
1	1.786246000	3.260943000	-2.515385000
1	1.032961000	4.213781000	-1.174950000
8	0.109756000	-1.422680000	0.897692000
6	0.176777000	-1.128822000	2.257670000
1	0.727108000	-1.946657000	2.763530000
1	-0.834278000	-1.099748000	2.711151000
6	0.893366000	0.198341000	2.491687000
1	0.225072000	1.044945000	2.254874000
1	1.199815000	0.286766000	3.549319000
8	2.025290000	0.254287000	1.646322000

1	3.906307000	0.948125000	0.556174000

Int_4

E(scf)= −1205.89773431

8	-1.820781000	0.293861000	1.390070000
6	-1.341780000	-0.551496000	0.385748000
6	-2.569896000	-1.036790000	-0.403917000
8	-0.422721000	0.059455000	-0.394738000
8	-3.221006000	0.098165000	-0.977212000
6	-2.437961000	1.476404000	0.917061000
6	-3.119536000	1.322016000	-0.447221000
8	-3.596521000	2.266324000	-1.008302000
6	-1.484344000	2.665811000	0.927940000
1	-3.264301000	1.672738000	1.622768000
1	-0.661824000	2.497447000	0.216621000
1	-1.065845000	2.788829000	1.940435000
1	-2.026802000	3.581996000	0.648381000
6	-2.266169000	-1.998590000	-1.530543000
1	-3.256542000	-1.502268000	0.323156000
1	-1.605581000	-1.518965000	-2.271612000
1	-3.199950000	-2.285435000	-2.039912000
1	-1.785247000	-2.907598000	-1.134319000
50	1.277620000	-1.083936000	-0.777169000
8	3.021206000	1.395926000	0.911827000
6	2.819675000	1.620556000	-0.290443000
6	3.151921000	2.968054000	-0.878839000
8	2.320470000	0.767315000	-1.111276000
1	2.206949000	3.499880000	-1.085138000
1	3.675255000	2.843020000	-1.839539000
1	3.758146000	3.558901000	-0.178149000
8	-0.773721000	-1.686195000	1.014004000
6	-0.214039000	-1.544864000	2.308486000
1	0.179415000	-2.545597000	2.545904000
1	-0.994282000	-1.284696000	3.041847000
6	0.897679000	-0.514470000	2.388226000
1	0.488935000	0.497849000	2.244209000
1	1.358133000	-0.574678000	3.390196000
8	1.895494000	-0.775606000	1.408367000
1	2.479886000	0.081626000	1.312664000

Int_6

E(scf)= −1205.87700229

8	1.199612000	0.286800000	1.143650000
6	1.047066000	0.447806000	-0.236270000
6	2.435649000	0.746134000	-0.825505000
8	0.464311000	-0.600819000	-0.837488000
8	3.311085000	-0.341781000	-0.521564000
6	2.005754000	-0.811148000	1.521640000
6	3.097764000	-1.169604000	0.507589000
8	3.786517000	-2.137213000	0.658950000
6	1.162893000	-2.025633000	1.892698000
1	2.560124000	-0.474123000	2.415439000
1	0.591140000	-2.375363000	1.019178000
1	0.461848000	-1.736362000	2.692845000
1	1.810717000	-2.838547000	2.254836000
6	2.434676000	0.928180000	-2.326711000
1	2.834927000	1.647227000	-0.327946000
1	2.043148000	0.019156000	-2.809138000
1	3.460370000	1.109961000	-2.685283000
1	1.802831000	1.788218000	-2.602379000
50	-1.499750000	-0.139941000	-1.349778000

1	2.072803000	1.217462000	1.286488000

Int_5

E(scf)= −1205.89142099

8	-2.052195000	0.391039000	-1.221758000
6	-1.341740000	0.738626000	-0.065422000
6	-2.359001000	0.744374000	1.089736000
8	-0.317362000	-0.119861000	0.152818000
8	-2.922533000	-0.562382000	1.215623000
6	-2.599732000	-0.912317000	-1.206173000
6	-2.965217000	-1.421666000	0.192378000
8	-3.338559000	-2.547306000	0.360452000
6	-1.722725000	-1.911735000	-1.951726000
1	-3.568248000	-0.825943000	-1.730299000
1	-0.755448000	-2.020366000	-1.438523000
1	-1.549600000	-1.546434000	-2.977635000
1	-2.224273000	-2.890556000	-1.997644000
6	-1.790200000	1.121603000	2.439119000
1	-3.161267000	1.444871000	0.803436000
1	-1.030610000	0.385300000	2.751051000
1	-2.589548000	1.123999000	3.197241000
1	-1.340928000	2.126800000	2.392801000
50	1.514675000	0.568377000	0.836665000
8	1.964282000	-1.720655000	1.089780000
6	2.499184000	-1.769926000	-0.039398000
6	3.014737000	-3.063716000	-0.602932000
8	2.587720000	-0.705107000	-0.735598000
1	3.976993000	-2.900827000	-1.112030000
1	2.292275000	-3.426559000	-1.354200000
1	3.113411000	-3.818091000	0.189795000
8	-0.894336000	2.060145000	-0.220685000
6	-0.562923000	2.516233000	-1.515872000
1	-0.193561000	3.542374000	-1.363199000
1	-1.460416000	2.553536000	-2.155863000
6	0.496595000	1.693652000	-2.224425000
1	0.124783000	0.672365000	-2.403913000
1	0.712664000	2.174266000	-3.196663000
8	1.678923000	1.657354000	-1.435621000
1	2.258670000	0.949843000	-1.769407000

Int_7

E(scf)= −1205.87489056

8	-2.116750000	0.287326000	-0.277153000
6	-0.906895000	-0.092696000	0.314326000
6	-1.222688000	-0.981349000	1.530183000
8	-0.045683000	-0.675019000	-0.531214000
8	-2.036820000	-2.086338000	1.129220000
6	-2.911818000	-0.773601000	-0.770106000
6	-2.798490000	-2.072560000	0.032247000
8	-3.405231000	-3.051490000	-0.296838000
6	-2.674001000	-1.025080000	-2.255820000
1	-3.955304000	-0.444646000	-0.625606000
1	-1.649997000	-1.391641000	-2.422799000
1	-2.812599000	-0.080674000	-2.807014000
1	-3.392315000	-1.771486000	-2.628089000
6	0.012007000	-1.548205000	2.194689000
1	-1.811205000	-0.382656000	2.246846000
1	0.542231000	-2.192800000	1.476184000
1	-0.274928000	-2.146728000	3.074330000
1	0.686229000	-0.735068000	2.506149000
50	1.516361000	0.637478000	-0.977125000

8	-2.090936000	-2.077746000	-0.265558000
6	-2.344808000	-1.434070000	0.786780000
6	-2.779223000	-2.149404000	2.032214000
8	-2.202146000	-0.172292000	0.793789000
1	-3.394227000	-1.491479000	2.662984000
1	-1.873849000	-2.429907000	2.597347000
1	-3.320959000	-3.070707000	1.773891000
8	0.170622000	1.552371000	-0.421013000
6	0.248531000	2.694948000	0.419203000
1	0.096383000	3.578680000	-0.221165000
1	1.245363000	2.765922000	0.880143000
6	-0.812772000	2.656295000	1.505401000
1	-1.810084000	2.534611000	1.035529000
1	-0.639151000	1.773735000	2.146830000
8	-0.705172000	3.869752000	2.205410000
1	-1.325368000	3.862417000	2.944139000

TS_3

E(scf)= −1205.86857227

ν_{min}= −46.1064

8	-1.124477000	0.749118000	-0.795911000
6	-0.760807000	0.542959000	0.553350000
6	-1.957837000	0.910207000	1.458286000
8	-0.434902000	-0.747672000	0.770166000
8	-3.039440000	0.027248000	1.160813000
6	-2.441562000	0.395628000	-1.176971000
6	-3.240884000	-0.353068000	-0.101300000
8	-4.066007000	-1.177518000	-0.373829000
6	-2.390840000	-0.382832000	-2.477940000
1	-3.005782000	1.337368000	-1.339834000
1	-1.901355000	-1.359091000	-2.321603000
1	-1.817945000	0.189908000	-3.224733000
1	-3.406000000	-0.567973000	-2.858924000
6	-1.652537000	0.781745000	2.933250000
1	-2.278275000	1.940863000	1.222062000
1	-1.318691000	-0.243777000	3.152888000
1	-2.556790000	1.003703000	3.522523000
1	-0.854996000	1.485766000	3.216233000
50	0.902817000	-1.900580000	-0.252506000
8	2.257195000	-0.217567000	-0.886999000
6	2.886036000	-0.266333000	0.213329000
6	3.927791000	0.758418000	0.540119000
8	2.595767000	-1.169025000	1.048162000
1	4.697527000	0.329497000	1.198336000
1	3.422279000	1.573769000	1.086513000
1	4.373421000	1.169683000	-0.376762000
8	0.339701000	1.354695000	0.809951000
6	0.323207000	2.697388000	0.375630000
1	0.826257000	3.292627000	1.155954000
1	-0.703288000	3.087505000	0.268954000
6	1.056881000	2.887661000	-0.943144000
1	2.077900000	2.469023000	-0.856353000
1	0.532279000	2.321691000	-1.733797000
8	1.071613000	4.272710000	-1.192629000
1	1.480328000	4.431883000	-2.051621000

TS_4

E(scf)= −1205.85609641

ν_{min}= −169.0548

8	-0.717362000	0.070176000	-0.886511000
6	-0.381928000	0.878607000	0.961703000

8	2.628909000	0.116858000	0.898964000
6	3.140213000	-0.924837000	0.380043000
6	4.005895000	-1.820567000	1.216231000
8	2.885245000	-1.203092000	-0.820571000
1	4.627563000	-2.464407000	0.578451000
1	3.346259000	-2.457794000	1.830508000
1	4.626879000	-1.220199000	1.897734000
8	-0.284666000	1.117242000	0.738865000
6	-1.036002000	2.169413000	1.320120000
1	-0.437042000	2.580063000	2.148401000
1	-1.984512000	1.787736000	1.727532000
6	-1.319481000	3.257364000	0.297704000
1	-0.357997000	3.573624000	-0.161433000
1	-1.958004000	2.835064000	-0.500980000
8	-1.941914000	4.309801000	0.984388000
1	-2.210478000	4.988084000	0.353070000

Int_8

E(scf)= −1205.87172199

8	-0.859021000	-0.336038000	0.629112000
6	-0.686551000	-0.459500000	-0.809879000
6	-2.067212000	-0.654244000	-1.471115000
8	-0.085703000	0.653083000	-1.235941000
8	-2.968710000	0.354283000	-1.008713000
6	-2.172013000	-0.322696000	1.168937000
6	-3.100458000	0.539322000	0.306996000
8	-3.892278000	1.318246000	0.753414000
6	-2.098436000	0.163831000	2.599942000
1	-2.577911000	-1.354490000	1.156545000
1	-1.735957000	1.204585000	2.637785000
1	-1.415632000	-0.480397000	3.176403000
1	-3.097435000	0.137879000	3.059767000
6	-1.990806000	-0.553301000	-2.977358000
1	-2.472679000	-1.640124000	-1.181106000
1	-1.603862000	0.437048000	-3.259865000
1	-2.990613000	-0.698386000	-3.416582000
1	-1.306850000	-1.324333000	-3.363940000
50	0.818438000	1.698422000	0.314350000
8	2.217117000	0.005312000	0.768689000
6	3.004195000	0.333871000	-0.172914000
6	4.148367000	-0.563778000	-0.536405000
8	2.783138000	1.392177000	-0.818651000
1	4.946873000	0.006403000	-1.032285000
1	3.762948000	-1.317541000	-1.245128000
1	4.527966000	-1.086199000	0.353805000
8	0.135131000	-1.553187000	-1.061310000
6	-0.087937000	-2.763439000	-0.375682000
1	-0.038250000	-3.583121000	-1.112248000
1	-1.085076000	-2.803834000	0.096717000
6	0.968844000	-2.983703000	0.693671000
1	1.969837000	-2.920354000	0.221433000
1	0.905757000	-2.170963000	1.439666000
8	0.722819000	-4.250686000	1.252935000
1	1.361072000	-4.410965000	1.958096000

Int_9

E(scf)= −1205.85775399

8	0.762696000	-0.065375000	1.036213000
6	0.234802000	1.045874000	-1.041430000

6	-1.702306000	1.638605000	0.995614000
8	-0.259347000	-0.202623000	1.571019000
8	-2.791548000	0.747341000	0.887687000
6	-2.014044000	0.190542000	-1.378876000
6	-3.047759000	0.058714000	-0.248215000
8	-4.036026000	-0.612780000	-0.309277000
6	-2.309507000	-0.795465000	-2.501079000
1	-2.138653000	1.217372000	-1.790667000
1	-2.296625000	-1.833518000	-2.126630000
1	-1.543107000	-0.684828000	-3.285264000
1	-3.304199000	-0.615440000	-2.937680000
6	-1.840955000	2.362055000	2.327959000
1	-1.732037000	2.370221000	0.172394000
1	-1.804040000	1.632243000	3.151938000
1	-2.808142000	2.887629000	2.359268000
1	-1.028490000	3.095465000	2.457711000
50	0.148432000	-1.771667000	0.006855000
8	2.000597000	-0.810815000	-0.700267000
6	2.737113000	-1.139648000	0.296900000
6	4.149813000	-0.625559000	0.340792000
8	2.264958000	-1.831067000	1.215638000
1	4.769053000	-1.258337000	0.992520000
1	4.124456000	0.393559000	0.764579000
1	4.576065000	-0.567840000	-0.671899000
8	0.723726000	1.555187000	0.706056000
6	0.766321000	2.577003000	-0.280492000
1	0.451592000	3.542875000	0.152064000
1	0.111420000	2.304360000	-1.121417000
6	2.198762000	2.659758000	-0.769605000
1	2.867511000	2.836392000	0.097961000
1	2.461039000	1.682184000	-1.216568000
8	2.244397000	3.714341000	-1.694102000
1	3.104360000	3.715252000	-2.131208000

Int_{10}

E(scf)= −1205.88683317

8	2.436636000	-0.896519000	1.289944000
6	-3.267340000	-0.003463000	0.528161000
6	-2.122629000	-0.864965000	0.006338000
8	-3.253798000	0.638268000	1.537507000
8	-1.102521000	-0.808136000	0.997153000
6	1.163003000	-0.668771000	1.745189000
6	0.155925000	-0.848836000	0.616737000
8	0.493421000	-1.000398000	-0.547150000
6	0.976988000	0.727874000	2.359306000
1	0.887886000	-1.422061000	2.513862000
1	1.143191000	1.497819000	1.587627000
1	1.728000000	0.857067000	3.154664000
1	-0.033493000	0.852327000	2.782688000
6	-2.526556000	-2.307586000	-0.264466000
1	-1.743561000	-0.405193000	-0.921320000
1	-2.882550000	-2.788929000	0.661157000
1	-1.659976000	-2.866738000	-0.651363000
1	-3.329741000	-2.336924000	-1.015989000
50	2.945530000	-0.731784000	-0.690221000
8	2.204132000	1.390148000	-0.780033000
6	3.306888000	1.894867000	-0.386226000
6	3.395676000	3.382840000	-0.181649000
8	4.282064000	1.152344000	-0.138030000
1	4.432650000	3.726443000	-0.307860000

6	1.480819000	1.894268000	-0.820419000
8	0.281336000	-0.016975000	-1.646983000
8	2.634078000	1.092169000	-0.863100000
6	2.088503000	0.171318000	1.379185000
6	3.000936000	0.252102000	0.139058000
8	4.008927000	-0.377939000	0.005466000
6	2.640728000	-0.842933000	2.373958000
1	2.144283000	1.174106000	1.859985000
1	2.704993000	-1.845733000	1.917593000
1	1.965290000	-0.885412000	3.243787000
1	3.652491000	-0.569179000	2.712881000
6	1.604286000	2.924483000	-1.939302000
1	1.405369000	2.411773000	0.148234000
1	1.685290000	2.412874000	-2.911519000
1	2.513814000	3.524111000	-1.779675000
1	0.729875000	3.595477000	-1.954833000
50	0.055573000	-1.761501000	-0.040143000
8	-1.870714000	-1.020946000	0.620715000
6	-2.622596000	-1.363052000	-0.373649000
6	-4.079073000	-0.984406000	-0.294621000
8	-2.148565000	-1.944182000	-1.352651000
1	-4.672937000	-1.605391000	-0.980408000
1	-4.178036000	0.071009000	-0.602658000
1	-4.450348000	-1.077130000	0.737383000
8	-0.933737000	1.533472000	-0.695916000
6	-1.087493000	2.437064000	0.397544000
1	-0.809964000	3.461924000	0.096376000
1	-0.460087000	2.094169000	1.234147000
6	-2.546404000	2.392115000	0.804822000
1	-3.176531000	2.598907000	-0.084736000
1	-2.768588000	1.368554000	1.160856000
8	-2.705680000	3.364305000	1.803008000
1	-3.577354000	3.267786000	2.204847000

1	3.082488000	3.603843000	0.853870000
1	2.717896000	3.911286000	-0.867828000
8	-4.294271000	-0.056093000	-0.323338000
6	-5.451250000	0.702112000	0.014310000
1	-5.188768000	1.766583000	0.125529000
1	-5.864314000	0.351823000	0.974339000
6	-6.457048000	0.508872000	-1.102506000
1	-6.011826000	0.864047000	-2.054066000
1	-6.667560000	-0.574265000	-1.214624000
8	-7.599021000	1.238158000	-0.745949000
1	-8.261920000	1.152238000	-1.441624000
